# Methanogens predominate in natural corrosion protective layers on metal sheet piles

**DOI:** 10.1038/s41598-017-11244-7

**Published:** 2017-09-19

**Authors:** Nardy Kip, Stefan Jansen, Marcio F. A. Leite, Mattias de Hollander, Michael Afanasyev, Eiko E. Kuramae, Johannes A. Van Veen

**Affiliations:** 1 Department of Microbial Ecology, Netherlands Institute of Ecology (NIOO-KNAW), Droevendaalsesteeg 10, 6708 PB Wageningen, The Netherlands; 2 Deltares, Princetonlaan 6, 3584 CB Utrecht, The Netherlands; 3 Department of Geoscience & Engineering, Delft University of Technology, Stevinweg 1, 2628 CN Delft, The Netherlands

## Abstract

Microorganisms are able to cause, but also to inhibit or protect against corrosion. Corrosion inhibition by microbial processes may be due to the formation of mineral deposition layers on metal objects. Such deposition layers have been found in archaeological studies on ancient metal objects, buried in soil, which were hardly corroded. Recent field investigations showed that natural mineral deposition layers can be found on sheet piles in soil. We investigated the microbial communities of these deposition layers and the adjacent soil. Our data, from five different sampling sites, all show striking differences between microbial communities of the deposition layer versus the adjacent soil over the depth profile. Bacterial species dominated in top soil while archaeal sequences increased in abundance with depth. All mineral deposition layers from the steel surface were dominated by Euryarchaeota, of which almost all sequences were phylogenetically related with the *Methanobacteria* genus. The mineral layer consisted of carbonate precipitates. Based on 16S rDNA gene sequencing data we hypothesize that the methanogens directly extract electrons from the metal surface, thereby, initially inducing mild corrosion, but simultaneously, inducing carbonate precipitation. This, will cause encrustation of the archaea, which drastically slow down their activity and create a natural protective layer against further corrosion.

## Introduction

Corrosion of materials is due to (bio) electrochemical reactions of these materials with their environment. Many metal constructions, such as those in harbors and waterways are of great economical and societal importance and, thus, corrosion can have enormous societal and economical effects. Present corrosion control strategies are often expensive, not always effective and environmentally unfriendly. Over time metal constructions need to be renovated or replaced, due to corrosion damage. In recent years it became clear that microbes may play key roles, not only in causing corrosion, so called microbially induced corrosion, or MIC, but also in inhibiting or protecting against corrosion, also referred to as MICI^1,2^.

Therefore there is an increasing interest in the micro-organims involved in corrosion in order to develop efficient and environmentally friendly corrosion control strategies. However, information on the composition and role of microbial communities related to corrosion and corrosion inhibition on different materials and in different environments is scarce.

Laboratory studies showed that certain single species biofilms can protect against corrosion^3–6^. Three possible mechanisms of microbially influenced corrosion inhibition (MICI) are^1,2,7^: 1) removal of corrosive substances which react with the metal surface, e.g. oxygen consumption by aerobic respiration. 2) growth inhibition of corrosion causing microbes, e.g by antimicrobial production. 3) formation of a protective layer, e.g. by the overproduction of extracellular polymeric substances (EPS). In multispecies biofilms a combination of the different mechanisms is to be expected. Remarkably, there is an increasing number of contradictory reports on both accelerating and inhibiting actions of the same functional group of microorganisms, such as sulfate reducers^8^, iron reducers^9^ and methanogens^10^ on the corrosion process. These studies showed that there could be a strong species specificity for either MIC or MICI, but also *in situ* conditions may play a role in the metabolism of different organisms and so in their reaction towards metal surfaces.

Most microbiological corrosion (inhibition) studies have so far been performed in controlled laboratorium experiments with particular attention to sulphate reducers or single species biofilms on metal coupons. However, these experiments do not allow for a proper understanding of the mechanisms of corrosion inhibition under natural conditions.

There are interesting observations in archaeological studies that do show that natural protective layers can be found at field conditions. At several archaeological sites well-preserved iron objects of up to 2000 years old were found even at highly corrosive soil conditions. All objects showed a highly adherent and compact coating consisting of mainly iron phosphates, which is thought to be due to microbial activity^11–13^. This mineral deposition layer is hypothesized to be closely related to a microbial biofilm. Phosphate and carbonate precipitation is common in soil environments^14,15^. Nevertheless, industrial phosphate coatings have never shown the same protection properties as the bacterial phosphate precipitate layers^16^. It is therefore important to investigate these naturally produced coatings.

Sheet piling is used as a reinforcement of waterways and dikes. In the Netherlands sheet piles are replaced every 50 to 100 years to ensure the maintenance of adequate safety against flooding. Recent renovations of sheet pilings in The Netherlands revealed similar adherent and compact coatings as found in archaealogical studies which could act as natural protective layers against corrosion. We had the unique opportunity to sample 50–100 year old metal sheetpiles, that were removed during renovations, at different places in the Netherlands. Here we report on our investigations of the microbial communities of soil and steel deposit layers over a depth profile in the soil. Microbial communities were analysed using 454 pyrosequencing based on the 16S rDNA gene. In combination with information on physicochemical parameters we tried to get a better understanding of possible processes that could lead to the production of natural protective layers.

## Results

### Sheet piles: general observations

Visual inspection of the sheet piles showed only corrosion at the surface level. At site HI corrosion was detected on a nearby sheet pile. Thickness measurements did not show significant thickness loss (data not shown), indicating no serious corrosion had taken place. All sheet piles inpected showed some signs of deposition layers. At the sites NLL and WK a more continuous and thicker layer was found than at sites GB, AZ and HI. In most cases the deposition layers were a few mm thick and usually it could be scraped off or even peeled off (Figure S1). Samples were taken from the deposition layer (DL), the attached soil (AS) and bulk soil (BS) at different depths. The microbial communities were investigated and where possible physicochemical analyses were performed on the samples, see Table S1.

### Mineral deposition layer analysis

The thick mineral deposition layer found in WK made it possible to carry out microscopic analyses. The microscopic analysis revealed a highly dense layer on the metal side and a more porous layer on the soil side of the sample (Figure S2). The dense layer had a higher concentration of iron rich minerals, most probably limonite, which is a mixture of hydrated iron oxide-hydroxides (FeO(OH)·nH_2_O). In the more porous layer sand grains and quartz crystals (SiO_2_) were visible. In between the sand grains carbonate minerals were detected, most probably calcite (CaCO_3_). X-ray crystallography (XRD) analysis showed the presence of quartz, wustite (FeO), calcite, siderite and chukanovite (Fe_2_(CO_3_)(OH)_2_) in the mineral layers of GB and NLL (data not shown).

### Community analysis: Archaea vs Bacteria

**Figure 1:**
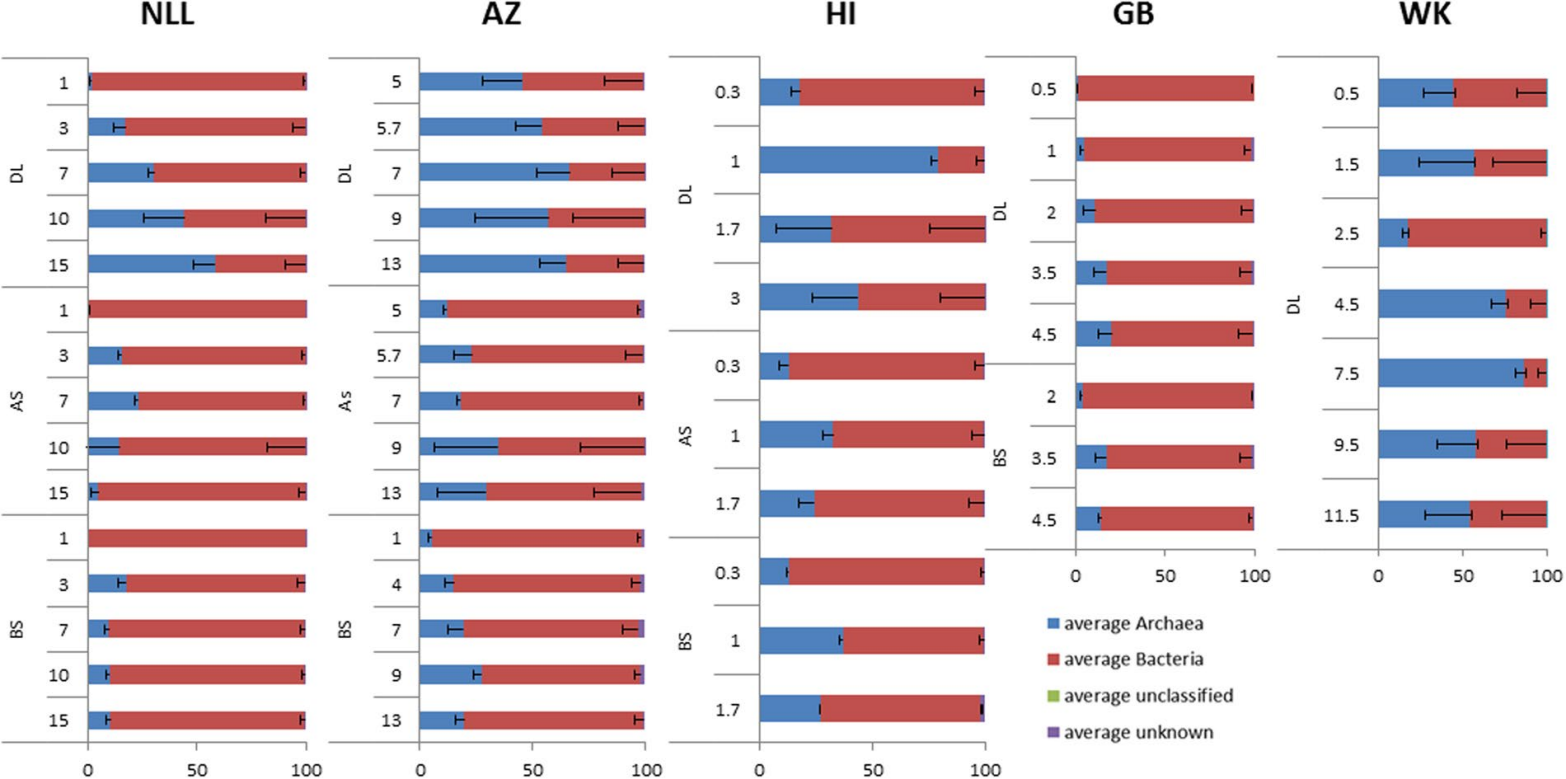
Archaeal and bacterial relative abundance (1 = 100% of all sequences) at the different sites. AZ = Amsterdam Zeeburgertunnel, NLL = Nieuwlekkerland, HI = Hollandse IJsselkade, GB = Giessenburg, WK = Westerkade. DL = deposition layer, AS = attached soil, BS = bulk soil.

Taxonomic classification of the 454 amplicon data revealed microbial communities that differed considerably between the different layers, *i.e*. the deposition layer (DL), the attached soil (AS) and the bulk soil (BS). Up to 2% of all sequences could not be classified taxonomically. The BS and AS samples were predominated by bacterial sequences, but in general, the archaeal sequence abundance increased with depth (Fig. 1). DL samples of all sites showed a high abundance of archaeal sequences, in particular the sites with a thick continuous deposition layer such as in the WK sample where archaeal sequences abundance was found to be as high as 87% of all sequences. At site NLL an increase in relative abundance of archaeal sequences was observed along the depth profile from 2% to 58% in DL versus a maximum of 17% in the BS. At site AZ the DL showed an increase in archaeal sequence abundance of 46 to 65% versus 5 to 20% in the BS. At the sites NLL and AZ a significant difference was observed between the community composition in the *DL* compared to the AS and the BS; in sites NLL (P = 0.009 and 0.02, respectively), AZ (P = 0.000). Also at sites HI and GB the same trends were observed. Figure 1 shows a clear decrease of archaeal sequence abundance with distance from the sheet pile.

### Community analysis: from class to genus taxonomic level

**Figure 2:**
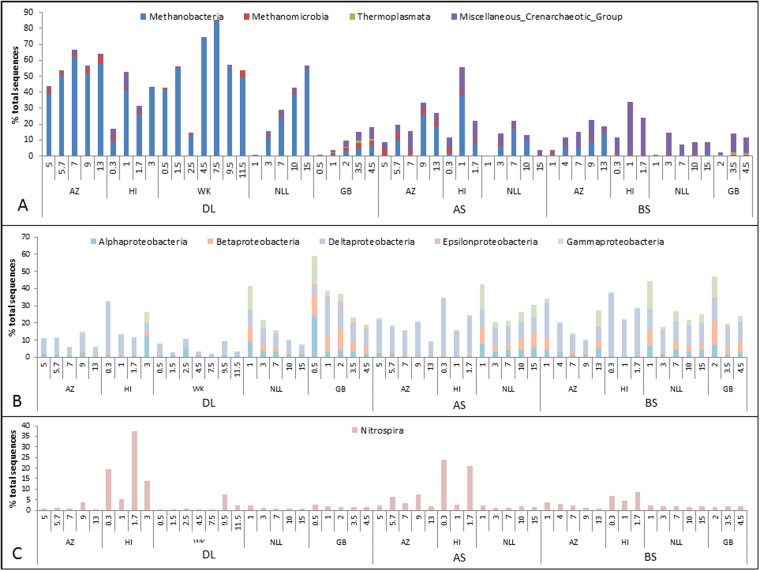
Most abundant phyla. AZ = Amsterdam Zeeburgertunnel, NLL = Nieuwlekkerland, HI = Hollandse IJsselkade, GB = Giessenburg, WK = Westerkade. DL = deposition layer, AS = attached soil, BS = bulk soil.

The core classes of the archaeal domain included *Methanobacteria* and the miscellaneous crenarchaeotic group (MCG) belonging to the *Thaumarchaeaota* and of the bacterial domain *Nitrospira*, *Alpha*-, *Beta*-, *Gamma*- and *Deltaproteobacteria*, *Coriobacteriia*, *Dehalococcoidia* and *Clostridia* were among the most abundant classes (Fig. 2, Figure S3 and Table S2). All deposition layers showed a high relative abundance of the archaeal class *Methanobacteria*, of up to 85%. The *BS* samples showed higher abundance of the Miscellaneous Crenarchaeotic Group (MCG). The bacterial phylum of *Proteobacteria* was found in all samples and the classes of *Alpha-, Beta-, Gammaproteobacteria* were rather evenly distributed among the different layers. The *Deltaproteobacteria* showed the highest relative abundance (2–36%) of all bacterial classes and their relative abundance increased with distance from the sheet pile; bulk soils having the highest abundance.

**Figure 3:**
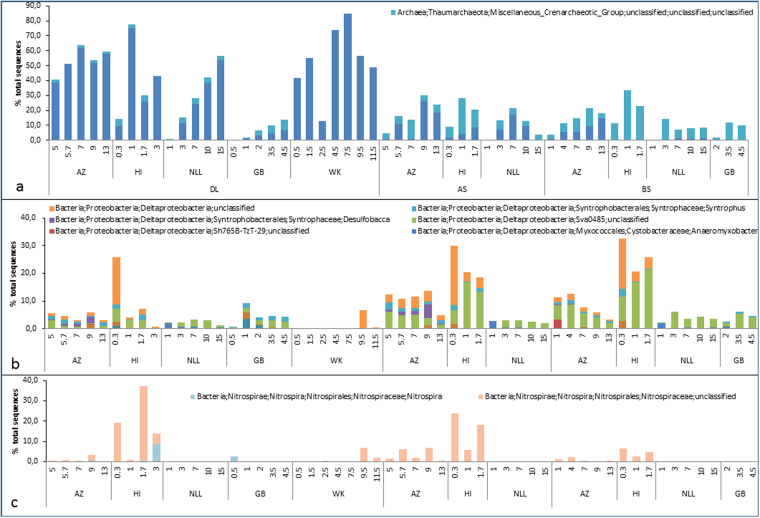
Most abundant genera. AZ = Amsterdam Zeeburgertunnel, NLL = Nieuwlekkerland, HI = Hollandse IJsselkade, GB = Giessenburg, WK = Westerkade. DL = deposition layer, AS = attached soil, BS = bulk soil.

Looking at deeper phylogenetic classification many OTUs could not be classified to a family or genus level (Fig. 3, Figure S4 and Table S3). The high relative abundance of archaea in the deposition layer is attributed largely to the high abundance of only one single genus; the *Methanobacterium* genus, which belongs to the phylum of *Euryarchaeota*. This genus showed relative abundances of up to 85% of the total prokaryotic sequences and was found especially abundant in the deposition layers. It represented almost all archaeal sequences of most deposition layer samples. At most sites the relative abundance of *Methanobacterium* increased with depth. *Methanobacterium* was found in lower abundance in the attached soil samples and was found in (very) low abundance in the bulk soil samples.

**Figure 4:**
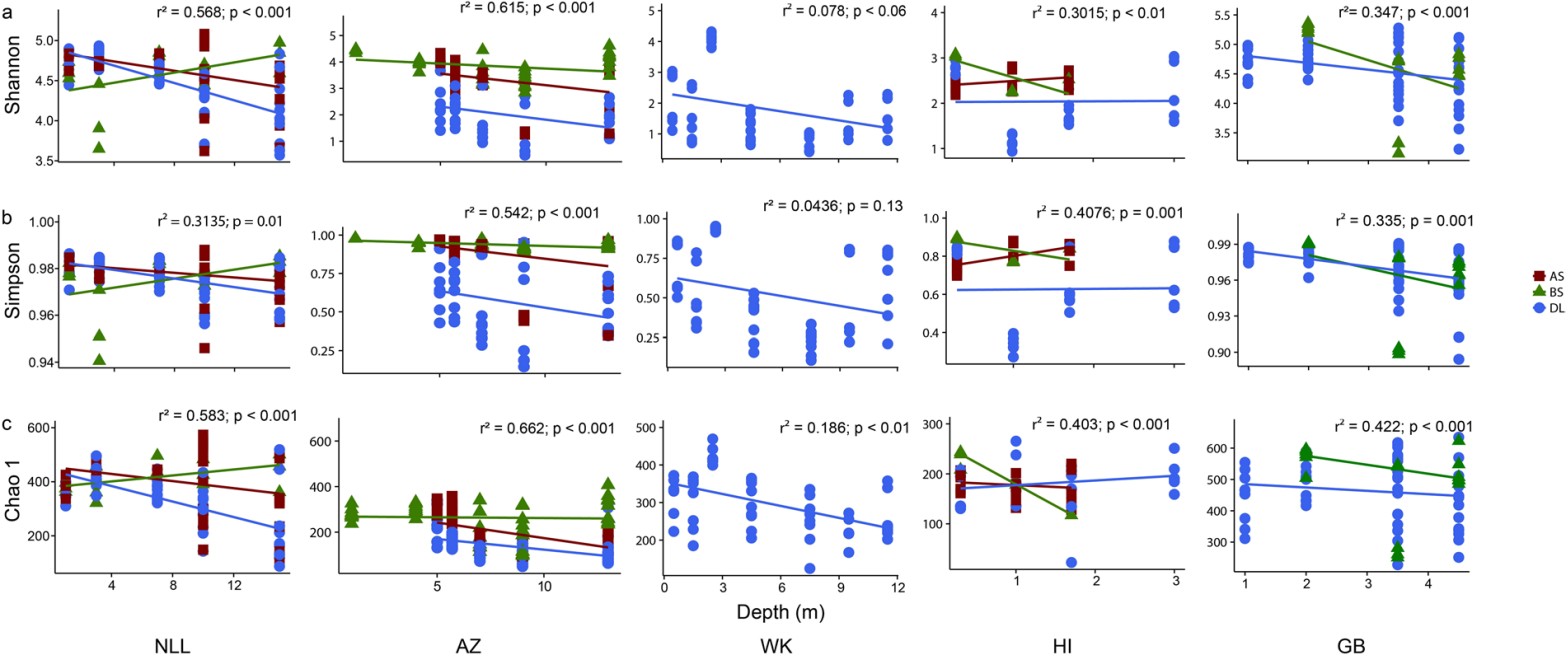
Shannon, Simpson and Chao1 indices for all the different sites. AZ= Amsterdam Zeeburgertunnel, NLL= Nieuwlekkerland, HI= Hollandse IJsselkade, GB= Giessenburg, WK= Westerkade. Blue dot= deposition layer, red box = attached soil, green triangle = bulk soil. Lines correspond to linear models of diversity indices according to depth for each sampling type. Coefficients of linear regression (r^2^) refer to the amount of variance explained by the model  with it correspondent value of significance (p-value).

Deltaproteobacterial sequences were found in all layers and at all sites, except WK. Many OTUs that were classified to the *Deltaproteobacteria* cannot be classified to a class or genus level (Fig. 3). OTUs classified to the Sva0485 group were abundantly (up to 21%) found in all samples. *Spirochaeta* were found at all the sites, except WK and represented 1–4% of the community in all the different layers. Of the *Nitrospira* class most OTUs were classified to the family of *Nitrospiraceae*, of which most OTUs could not be further classified, but some were classified to the *Nitrospira* genus.

### Diversity analysis

To characterize the diversity of the microbial communities of the different layers at all sites diversity indices, i.e. Shannon (H), Simpson (1-D) and Chao-1 were calculated in BiodiversityR^17^ (Fig. 4). We checked the effect of depth in the diversity indices via linear models and evaluated the fitness of the different models for each region by r² value. The Shannon index decreased in depth at most sites, with the largest decrease in the DL. The Simpson index showed the same trend as the Shannon index for the deposition layers. The Simpson and Shannon indices represent richness and evenness of a community and the low numbers in the DL indicated that only a few highly abundant OTUs dominated the microbial community. The Chao estimator (richness index) did not show the same trend indicating that there is still a considerable variety of OTUs in all samples. We report a significant decreasing associated with depth in the deposition layer (DL) in the regions NLL, AZ and WK while both HI and GB presented an oscilation in their community dominance and evenness. According to the regression coefficient, the depth explained the majority of differences in the diversity indices for AZ (54.2–66.2%) and NLL (31.3–56.8%) regions, while in WK region depth had low, but significant explanatory power (4.3–18.6%). In the other regions the depth explained 30.2–42.2% of the diversity indicesvariability. Therefore, the influence of depth in the microbial community diversity might be more relevant according to the region. In general, both attached soil (AS) and bulk soil (BS) did not differ in their diversity indexes along depth.

**Figure 5:**
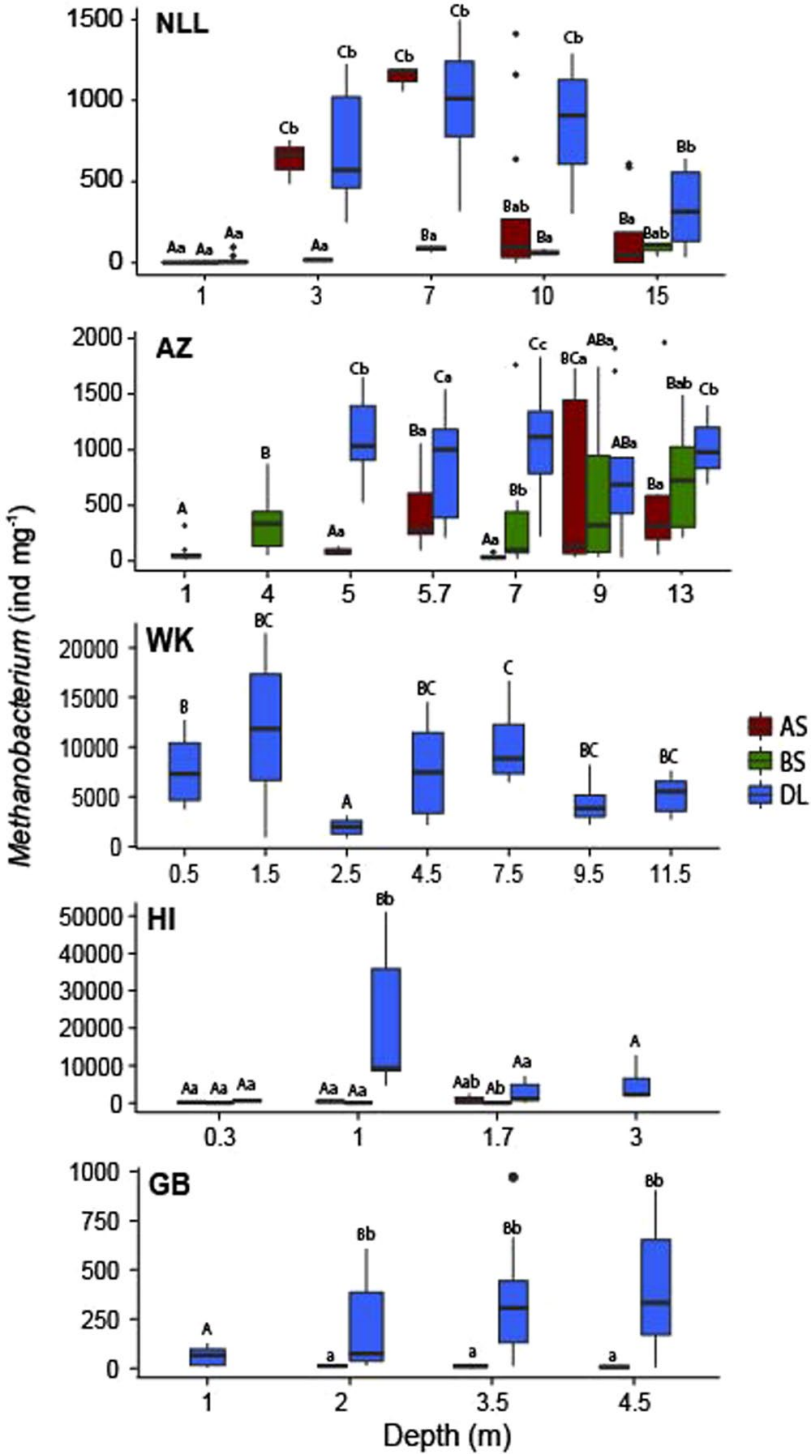
Abundance of Methanobacterium in the different sites. AZ = Amsterdam Zeeburgertunnel, NLL = Nieuwlekkerland, HI = Hollandse IJsselkade, GB = Giessenburg, WK = Westerkade. Lines and boxes represent medians and 25–75 percentiles of Methanobacterium abundance, whiskers are the maximum and minimum. Boxes followed within by the same capital letter did not differed according to depth within each layer (BS, AS and DL) while boxes followed by the small letter within the same depth did not differ between layers (BS, AS and DL). We applied a Tukey-Kramer multiple comparison test at 5% probability level in a generalized linear model in a negative binomial distribution.

### *Methanobacterium* associated with the deposition layer

Our analysis of *Methanobacterium* abundance revealed increase of *Methanobacterium* according to depth in the deposition layer, except in the WK region) (Fig. 5). We also noticed that this group of bacteria remained rare in bulk soil (BS) for all the regions until 7 m depth where they become more relevant in the AZ region. Moreover, the *Methanobacterium* abundance in the adjacent soil (AS) seemed to present a similar pattern for the first meter (1.7 m) in the HI region and increases at the depths of 3 and 7 m followed by a rapidly abundance decrease in deeper sampling points (10 and 15 m) in the NLL region. The abundance of *Methanobacterium* increases in sampling point 9 m dept but decreases in sampling point 13 m dept in AZ region. For the attached soil the *Methanobacterium* occurrence seemed strongly influence by regional varability rather than by depth.

**Figure 6:**
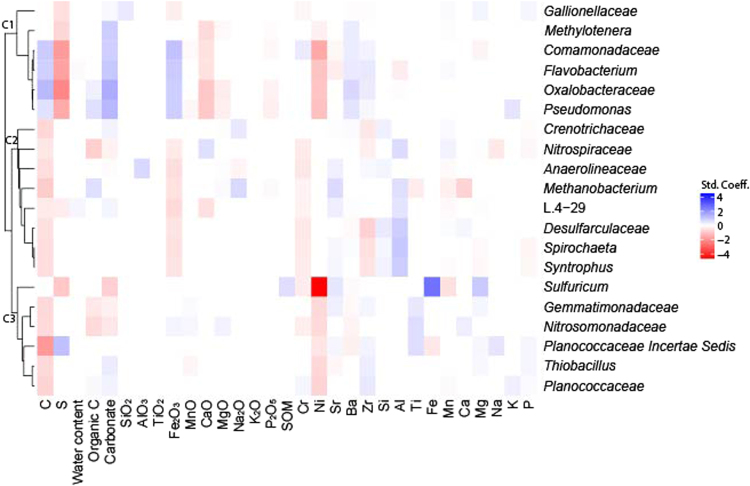
Standardized coefficients for the species distribution modelling between top 20 most abundant archaea and bacteria groups according to the environmental variables for both bulk soil and attached soil in site GB and NLL.

### Correlation analysis

When possible, the physicochemical analyses were performed on the BS and the AS samples (Table S1). There were large differences along the depth profiles of the different sites. For example, in site NLL pH, water content, calcium carbonate and organic carbon increased with depth, while they decreased with depth at site GB. Many other physicochemical factors showed variable patterns throughout the soil profile. Because the environmental factors also play a role in determining the microbial abundance, we evaluated the influence of the soil factors in both attached (AS) and bulk soil (BS) of both NLL and GB regions (Fig. 6). A total of 100 positive coefficients and 114 negative coefficients was calculated. The top 20 most abundant bacterial and archaeal groups responded mainly to the total C-content; only four groups (*Comamonadaceae*, *Flavobacterium*, *Oxalobacteraceae* and *Pseudomonas*) presented increase in abundance as a result of a higher soil C-content. The abundance of *Nitropiraceae*, *Gemmatimonadaceae* and *Nitrosomonadaceae* reduced in both soils and attached soils according to the organic C-content. Moreover, the 20 most abundant bacteria grouped in three main clusters according to their abundance and soil factors. The cluster C1 comprised of *Gallionellaceae*, *Methylotenera*, *Comamonadaceae*, *Flavobacterium*, *Oxalobacteraceae* and *Pseudomonas*, grouped the bacteria with a positive coefficients for C- content, Carbonate, Fe_2_O_3_, Ba and Zn, and negative coefficients for CaO and Ni. Cluster C2 grouped *Crenotrichaceae*, *Nitrospiraceae*, *Anaerolineaceae*, *Methanobacterium*, L.4–29, *Desulfurculaceae*, *Spirochaeta*, and *Syntrophus* which bacterial groups responded negatively to C, Fe_2_O_3_ and Cr, and positively to Sr- and Al-contents. Finally, the cluster C3 is comprised of *Sulfuricum*, *Gemmatimonadaceae*, *Nitrosomonadaceae*, *Planococcaceae* Incertae Sedis, *Thiobacillus* and *Planococcaceae* which appeared to decrease their abundance according to C-content and Ni-content (Fig. 6).

## Discussion

Microbes are able to inhibit corrosion, but the mechanisms underlying this process are not yet understood. Recent inspections of extracted sheet piles in the Netherlands showed that the metal sheet piles were not corroded and in many cases there was a mineral deposition layer present. In archaeological studies^2,11^ similar deposition layers have been found, that are naturally protective against corrosion and are thought to be the result of microbial biofilm formation. Aiming to understand better the role of microbes in the corrosion and corrosion protection processes we therefore studied the microbial communities of mineral deposition layers on sheet piles from different sites.

Remarkably the deposition layers showed a predominant abundance of archaea of up to 85% of the total bacterial and archaeal communities, whereas the communities of other samples of attached and bulk soil were dominated by bacteria. Even more remarkable is the fact that the archaeal community was dominated by a single genus of *Methanobacterium*. Most soil studies report that 0–10% of the prokaryotic sequences can be assigned to archaea^18,19^. Microbial community studies in soils are usually performed on the top 15–25 cm of the soil^20,21^, since this is the most active and microbially dense layer. There are few studies in which also samples from deeper layers, up to a meter, were included^20^. Eilers *et al*.^19^ sampled up to 150 cm and used the same 16S rRNA gene primers as in this study. They found that archaea only represented about 1% of all sequences and had relative abundances of 0–9% per sample, with the highest abundance in the deepest soil samples. In acidic forest soils the archaeal abundance was reported to be between 12–38%, based on clone libraries and terminal restriction length polymorphism (T-RFLP)^22^. Overall the relative abundance of Archaea in all these studies have never been shown to be as high as here.

The genus *Methanobacterium* was the most dominant in the deposition layers at all sites and represented up to 85% of the community. All cultivated isolates of this genus are methanogens. These methanogens are known to produce methane by oxidizing hydrogen, but are also able to use electrons from elemental iron (Fe^0^)^23,24^ or a metal electrode^25^. However the mechanism of electrosynthesis of methane by methanogenic rchaea is still unclear. Thus far, methanogens were often found on metal surfaces and are thought to cause corrosion. Usher and coworkers^26^ found a high abundance of methanogenic archaea, among which *Methanobacteria spp*., in rust tubercules. Some methanogenic rchaea are able to reduce iron-minerals, which can even inhibit methane production^27,28^.

Recently, new genomes and 16S rRNA amplicon sequencing expanded and reshaped the archaeal phylogenetic tree and new lineages have been proposed in the last years. The MCG or “Miscellaneous Crenarchaeota Group” that used to be designated to the *Thaumarcheaota* has now been proposed to be a fourth phylum, with the suggested name *Bathyarchaeota* ^29^ of the proposed TACK superphylum^30^. MCG archaea live in diverse habitats, including terrestrial and marine, hot and cold, surface and subsurface environments^31^. Here they were found to be the dominant archaeal group in the bulk soil samples. The role of MCG rchaea in geochemical cycles is so far not understood, but they have been suggested to be heterotrophic^32^ and able to degrade extracellular proteins^33^.

Many OTUs that were classified to the Deltaproteobacteria could not be classified to a class or genus level, indicating they could be novel species. The families of *Nitrospiraceae* are known as nitrite oxidizing bacteria and they were found abundantly in nearly all the samples. They were especially abundant at the HI site, where corrosion was found on nearby sheet pilings. *Nitrospira* have been mentioned in relation to corrosion; *Nitrospira moscoviensis* was isolated from a corroded iron pipe^34^ and grows optimally on nitrite and carbon dioxide, but can also grow under anoxic conditions using hydrogen as an electron donor and nitrite as electron acceptor.

Collectively, the microbial community analysis of the sheet piles from the different sites showed a clear enrichment of methanogenic rchaea in the deposition layer compared to a more diverse community in the attached soil or bulk soil. The metal surface is most probably the primary regulator controlling microbial functioning as it provides or takes up electrons which may make it a favorable habitat for microbes such as methanogens and sulfate reducers. Sulfate reducers are considered to be the main causers of corrosion and were detected to some extent in most samples. Nevertheless a high abundance of methanogens showed that the sheet pile surface clearly favors the growth of methanogens instead of sulfate reducers.

When steel corrodes it becomes covered by a mineral crust usualy consisting of iron oxide or hydroxide, also iron sulfides can be found under sulfate reducing conditions. The mineral crust can be electroconductive and can facilitate the release of electrons, thereby stimulating the corrosion process. However, the deposition layer found in this study is composed of different types of carbonates, such as calcium carbonate and iron carbonate. Carbonate precipitation is known to be a common process in soils. There are two mechanisms of carbonate precipitation: direct and indirect precipitation. A number of bacteria can directly induce carbonate minerals^35–37^. At indirect carbonate precipitation, the microorganisms do not influence the precipitation, it is only a side effect of their influence of the environment due to their metabolism, e.g. uptake or production of CO_2_ or pH change.

Microbially influenced carbonate precipitation is already used in the construction industry in ‘self healing concrete’^38^ and as well in conservation of copper objects^39^. In some cases, water treatment plants try to induce a small amount of calcium carbonate deposition (scaling) because it coats the insides of pipes and protects against corrosion^40^.

In archaeological studies carbonate and phosphate layers also were found on uncorroded metal objects^11,12^. These layers were thought to be naturally protective against corrosion. However, chemically produced phosphate layers never showed the same protective effect as the microbially produced layers.

Iron carbonate layers have also been reported in corrosion studies using iron coupons that were immersed in a methanogenic *Methanobacterium* culture^10^. In anaerobic waste water treatments methanogens were shown to induce precipitation and their composition and activity depends on the waste water composition^41^. Iron carbonate and iron phosphate layers have also been detected in corrosion experiments with a nonhydrogenotrophic nitrate reducing bacterium^42^.

Some methanogenic archaea, among which *Methanobacterium*, have been shown to be able to use electrons directly from metal or Fe^0^ also refered to as electromethanogenesis^24,43,44^. Nevertheless the underlying molecular mechanisms of direct electron uptake are still unknown. Recent experiments with cell free culture medium also demonstrated that cell-derived free enzymes from the electromethanogenic archaeon *Methanococcus maripaludis* can mimic direct extracellular electron transfer during Fe(0) corrosion and microbial electrosynthesis^45^. Also *Methanobacterium palustre* was shown to be able to perform electromethanogenesis^46^.

We hypothesize that the deposition layers are formed due to electrical microbially influenced corrosion by methanogens, consuming electrons from the iron and carbon dioxide from the soil. The carbon dioxide depletion and pH increase indirectly cause calcium carbonates to precipitate. The metal sheet pile provides the surface derived electrons at low redox potential to the methanogens, as it does for sulfate reducers in the electrical microbially influenced corrosion (EMIC) process^47,48^. In contrast to the electroconductive iron sulfate crust produced by sulfate reducing bacteria the carbonate layer as found here is not electroconductive and therefore corrosion stops or is slowed down when the microbes get entrapped in the layer and they are not anymore in direct contact with the metal surface. This entrapment can also be found in caves where calcium carbonate precipition is mainly due to microbial activity which induce deposition^49,50^. There the carbonate accumulation leads to entrapment of the bacteria in the mineral deposits, which drastically decreases their activity.

Since our 16S rDNA gene data show methanogenic rchaea in abundance in the deposition layer, we assume they are key to the production of this layer. Our hypothesis can only be proven when a culture is obtained and tested in laboratory conditions. There are other possible explanations for the occurance of the mineral deposition layer, for example the collaboration or succession of a multispecies community with metabolic activities resulting in the formation of precipitates or possibly methanogens were enriched while using the hydrogen produced by (a)biotic reactions.

The highly dense layer found in the microscopy experiments might indicate to the presence of a methanogenic biofilm on the metal surface. This biofilm may have also influenced the carbonate precipitation rates by higher local differences and the different polymeric substances have also been shown to influence precipitation^51^. In anaerobic sludge reactors carbonate precipitation was also shown to take place in the methanogenic layer of the granules^41^. A recent study^52^ showed that a *Vibrio neocaledonicus* biofilm on metal was able to increase corrosion resistance by 60-fold. The inhibitory effect was caused by the formation of the Fe-EPS complexes which strengthen over time.

## Conclusions

We found pronounced differences in microbial community structure between soil and mineral layers on the sheet piles along depth. Based on 16S rDNA gene sequencing data, *Methanobacterium* was found to be highly abundant in the deposition layers of the sheet piles. The layer consisted of carbonate precipitates and we hypothesize that the methanogens are able to directly extract electrons from the metal surface, thereby, initially, inducing corrosion, but at the same time the methanogens induce the precipitation of carbonates so that they get encrusted in the mineral deposition layer, which in turn, drastically slows down their activity forming a natural protective layer against further corrosion. Future research on isolated cultures from these samples is needed in order to test the proposed hypothesis.

## Experimental Procedures

### Sample collection and physicochemical analysis

Samples were taken immediately after pulling out the sheet piles from their original locations as part of a renovation project. Table 1 shows an overview of locations from where piles were sampled. All sheet piles were vertically placed. Only underground samples were collected from the metal sheet piles, meaning soil was present on both sides of the metal sheet pile. At some sites the upperpart of the sheet pile was in contact with water. In these cases samples were only taken from a depth where the sediment (soil) started to ensure a comparable situation for all samples. See Figure S1 for a schematic overview. The underground part of the sheet pile did not show many signs of corrosion, but different deposition layers were visible at different depths. After the extraction of the sheetpile from the soil, samples were taken from the deposition layer, the attached soil, which is soil directly adjacent to the deposition layer and bulk soil at different depths. From sites HI and GB two piles were sampled and from the other sites only one was extracted from the soil. Per depth samples were taken from different places of the sheetpile in duplo or triplo. Deposition layers were sampled by scraping off the adherent layer. When possible, layers were broken off. Samples were transported on ice and stored at −20 °C immediately.

**Table 1:** Site description. Length indicates the length of the sheet pile that was sampled.

Site name	Site	Length (m)	Sheet pile age (years)	Soil Material	layers sampled	Depth sampled (m)	Number of piles sampled	Number of samples per depth per layer
AZ	Zeeburgertunnel 52°21′46.9″N 4°57′26.3″E	13	30	peat	DL, AS & BS	5, 5.7, 7, 9, 13	1	3
NLL	Nieuwlekkerland 51°53′17.1″N 4°38′57.5″E	17	26	clay & peat	DL, AS & BS	1, 3, 7, 10, 15	1	3
WK	Westerkade 51°54′21.7″N 4°28′41.2“E	11.5	67	sand	DL	0.5, 1.5, 2.5, 4.5, 7.5, 9.5, 11.5	1	3
HI	Hollandse Ijsselkade 51°55′02.4″N 4°34′48.9″E	3.5	56	nd	DL, AS & BS	0.3, 1, 1.7	2	2
GB	Giessenburg 51°51′51.2″N 4°52′49.3″E	5	20–30	clay & peat	DL & BS	0.5, 1, 2, 3.5, 4.5	2	3

The sheet pile age is the approximately time that the sheet pile was in the soil. Samples: DL = deposition layer, AS = attached soil, BS = bulk soil.

The following physicochemical parameters were determined by Deltares (the Netherlands); total C and S (by flash combustion followed by CO_2_ analysis by an IR detector: Leco SC-632), SiO_2_, AlO_3_, TiO_2_, Fe_2_O_3_, MnO, CaO, MgO, Na_2_O, K_2_O, P_2_O_5_, soil organic matter (som), Cr, Ni, Sr, Ba, Zr, Si, Al, Ti, Fe, Mn, Ca, Mg, Na, K, P (all by XRF). The following parameters were determined by the Soil Science Department of Wageningen University and Research centre (the Netherlands): soil pH (CaCl_2_ extraction), nitrogen-, ammonium- and phosphorus concentrations (SFA-CaCl_2_), total dissolved organic carbon (DOC;SFA-TOC), carbonate concentration (Scheibler), Cl concentrations (H_2_0 extraction) and electric conductivity (EC).

### DNA extraction, 16S rDNA fragment amplification and sequencing

DNA was extracted from 0.25–0.3 g of (grinded) sample using the Powersoil DNA isolation kit (MOBIO laboratories, Carlsbad, CA) following the manufacturers protocol, with 30 Hz for 10-min bead beating instead of vortexing. DNA was isolated from 3 replicates per sample. The DNA concentration of each sample was measured using an ND-1000 spectrophotometer (Nanodrop, Wilmington, DE). Amplicons for barcoded pyrosequencing were obtained by PCR amplification of the V4 region of the 16S rRNA gene using the general prokaryotic primers F515 (5′-GTGCCAGCMGCCGCGGTAA-3′) and R806 (5′-GGACTACVSGGGTATCTAAT-3′). The forward primer contained multiplexed identifiers: the Roche 454-A adapter, a 10-bp barcode and a GT linker and the reverse primer contained the Roche 454-B adapter and a GG linker. PCR reactions were performed on 2–4 ng of sample DNA in a PCR mix containing 1 unit Taq polymerase (Faststart, ROCHE Indianapolis, IN), 1x buffer, 2 mM dNTPs (Invitrogen, Carlsbad, CA) and 2.5 µM of each primer. Denaturation was initiated at 95 °C for 5 min, followed by 25 cycles of: 95 °C 30 sec, 53 °C for 1 min and 72 °C for 1 min, with a final extension step at 72 °C for 10 min. Each sample was amplified in four replicates (25 μl). Two replicates were pooled to 50 μl PCR product and cleaned using a QIAquick PCR Purification Kit (Qiagen, Valencia, CA). Amplicons from different replicated samples were pooled in equimolar concentration and sequenced on a Roche 454 FLX Titanium platform (Macrogen Inc, South Korea) using the manufacturer’s protocols (Roche, Brandford, CA).

### 16S rRNA partial gene pyrosequencing analysis

The sequence data were processed using a Snakemake workflow^53^ in mothur version 1.33.2^54^ following the SOP for 454 data. The flowgrams were demultiplexed (mismatch barcode:2, mismatchprimer:3, size: 390 bp) and corrected using the shhh.flows command, which is the mothur implementation of the original PyroNoise algorithm^55^. The sequences were aligned to the bacterial reference alignment (http://www.mothur.org/wiki/Silva_reference_alignment) which is based on the SILVA 119 release of the SSURef database^56^. Chimeras were removed using the chimera.uchime command^57^. OTUs were formed using the dist.seqs command and average neighbor clustering, with a phylotype being defined at the 97% sequence similarity level. Taxonomic classification was performed by retraining the RDP classifier with the Silva reference files. Samples <100 sequences were discarded from further analysis. The numbers of reads per samples and the Good’s coverage are listed in Table S4. Diversity indices Simpson, Shannon, Chao1 were calculated using the package BiodiversityR^17^. The 454 FLX Titanium flowgrams (sff files) have been submitted to the EBI (accession no. PRJEB9787).

### Statistical analyses

The soil microbial community (bacteria and archaea) datasets presented an overdispersed variance, therefore, we applied a generalized linear model (GLM) based on negative binomial distribution to investigate the effects of the deposit layers and the adjacent soil on microbial structure. In order to avoid sequencing bias common in next-generation sequencing platforms^58,59^, we decided to use the total number of reads per sample as a covariance effect in our generalized linear models. The effect of both depth and position (bulk soil, attached soil and deposition layer) in the microbial community was evaluated by the Wald’s test (W), and the multiple comparisons were performed with Tukey-Kramer test.

Moreover, we investigated the microbial community interaction with environmental factors by applying the Species Distribution Modelling (SDM)^60,61^, which allowed us to obtain the coefficient of each environmental variable in determining the population number. This modelling was performed in a negative binomial distribution and we adopted the LASSO-penalised fit criterium to select only the environmental variables relevant to explain the population distribution (minimizing the Bayesian Information Criterium)^62^. In order to find clustering patterns in the microbial response to the soil factors we applied a hierarchical^63^. The R environment (R Development Core Team 2007) and “mvabund” libraries were used for the multivariate analyses and the species distribution modelling^64^, “multcomp” for multiple comparisons, and “pvclust” for the hierarchical clusterization^63^.

### Mineral deposition layer analysis

Samples from the mineral deposition layer of site WK were embedded in epoxy and slices of 30 µm were analysed by light microscopy, X-ray crystallography (XRF) and SEM/EDXA by SGS Intron b.v., the Netherlands.

### Accession codes

European Nucleotide Archive study accession no. PRJEB9787.

## Electronic supplementary material

Supplementary Material

Supplementary Table S1

Supplementary Table S2

Supplementary Table S3
