# Supplementary material for: Methanogens predominate in natural corrosion protective layers on metal sheet piles
Source: Sci Rep. 2017 Sep 19;7:11899. doi: 10.1038/s41598-017-11244-7 (PMC5605657; doi:10.1038/s41598-017-11244-7)
Supplement: Supplementary file 1 — Supplementary Material [file 41598_2017_11244_MOESM1_ESM.pdf]

Methanogens predominate in natural corrosion protective layers on metal sheet piles

Nardy Kip[1], Stefan Jansen[2] , Marcio F.A. Leite[1], Mattias de Hollander[1], Michael Afanasyev[3], Eiko E. Kuramae[1] and Johannes A. van Veen[1*]

[1]Department of Microbial Ecology, Netherlands Institute of Ecology (NIOO-KNAW), Droevendaalsesteeg 10, 6708 PB, Wageningen, the Netherlands.

[2]Deltares, Princetonlaan 6, 3584 CB Utrecht, the Netherlands

[3] Department of Geoscience & Engineering, Delft University of Technology, Stevinweg 1, 2628 CN Delft, the Netherlands

*Corresponding author: Hans van Veen H.vanVeen@nioo.knaw.nl. Department of Microbial Ecology, Netherlands Institute of Ecology (NIOO-KNAW), Wageningen, the Netherlands. Telephone: +31 (0)317 473 475

Supplementary Information

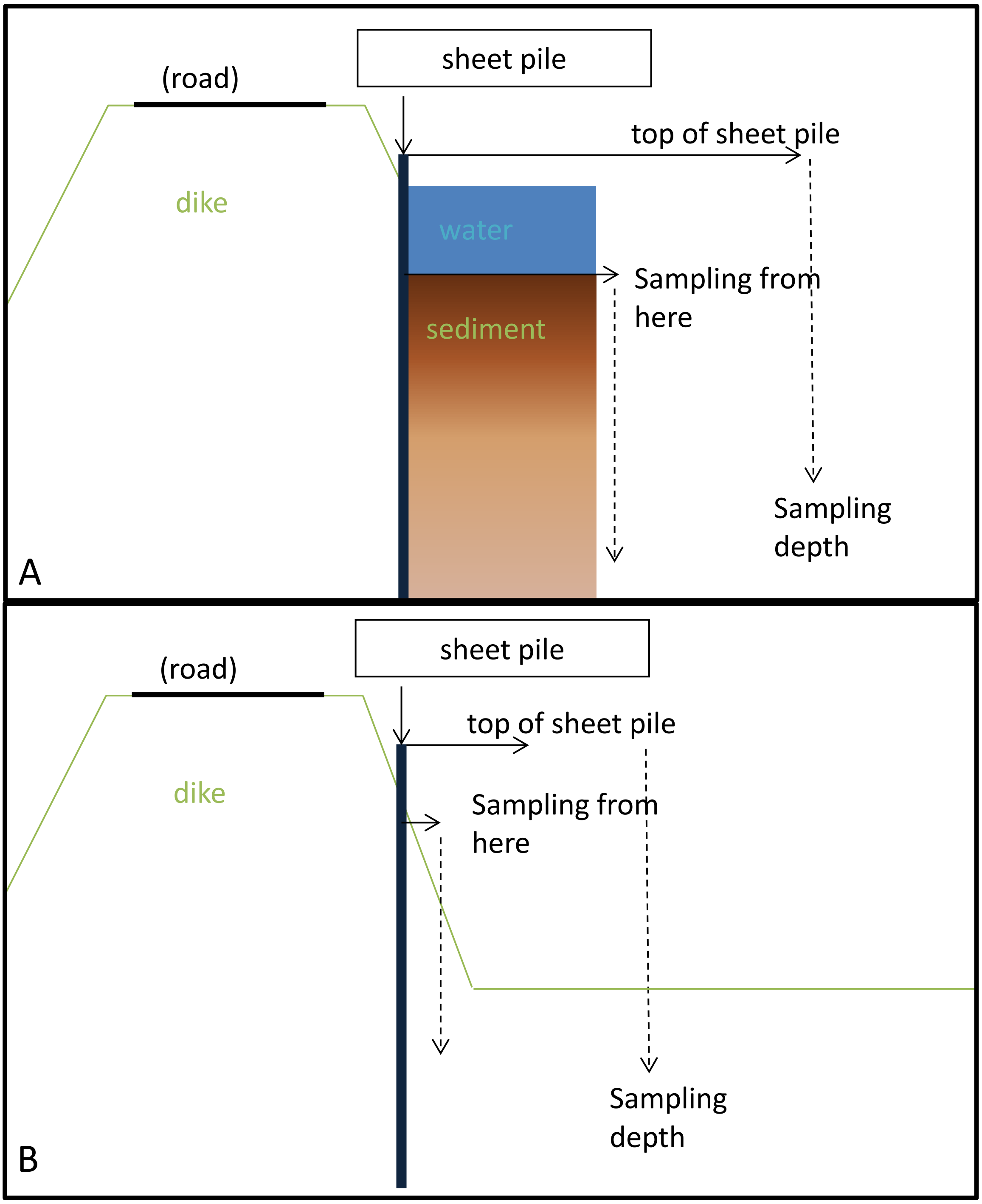
sheet pile
(road)
top of sheet pile
dike
water
Sampling from here
sediment
Sampling depth
A
sheet pile
(road)
top of sheet pile
dike
Sampling from here
Sampling depth
B

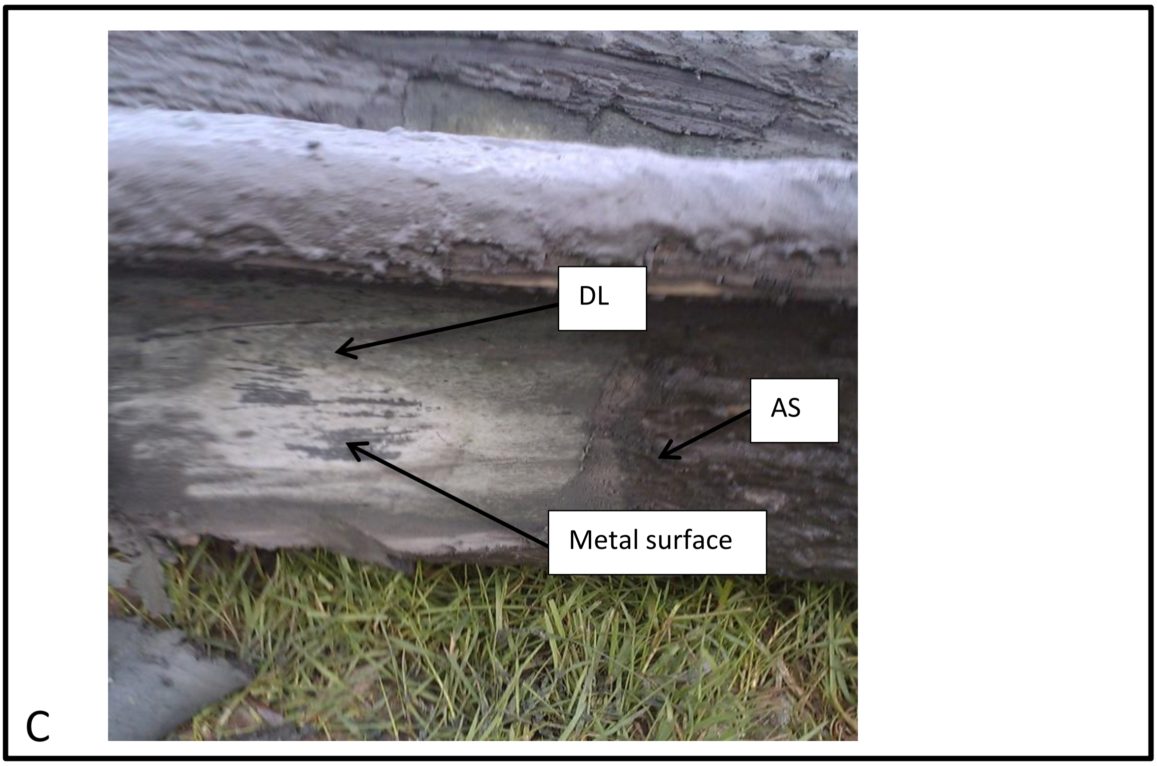

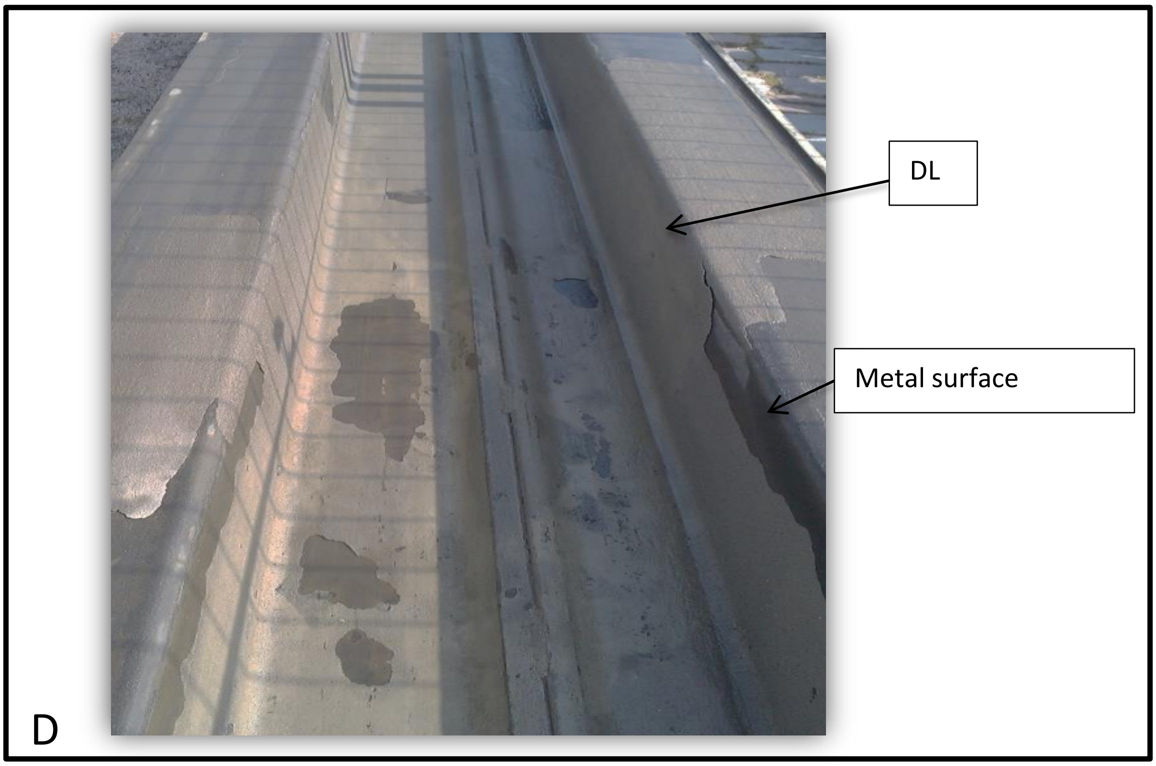

Figure S1. Schematic overview of how metal sheet piles are used to reinforce a waterway (A) and a dike (B). Photos of sheet pile with a mineral deposition layer. C: Sheet pile with attached soil and mineral deposition layer at Site NLL. D: very thick mineral deposition layer at site WK. DL=Deposition layer, AS=attached soil

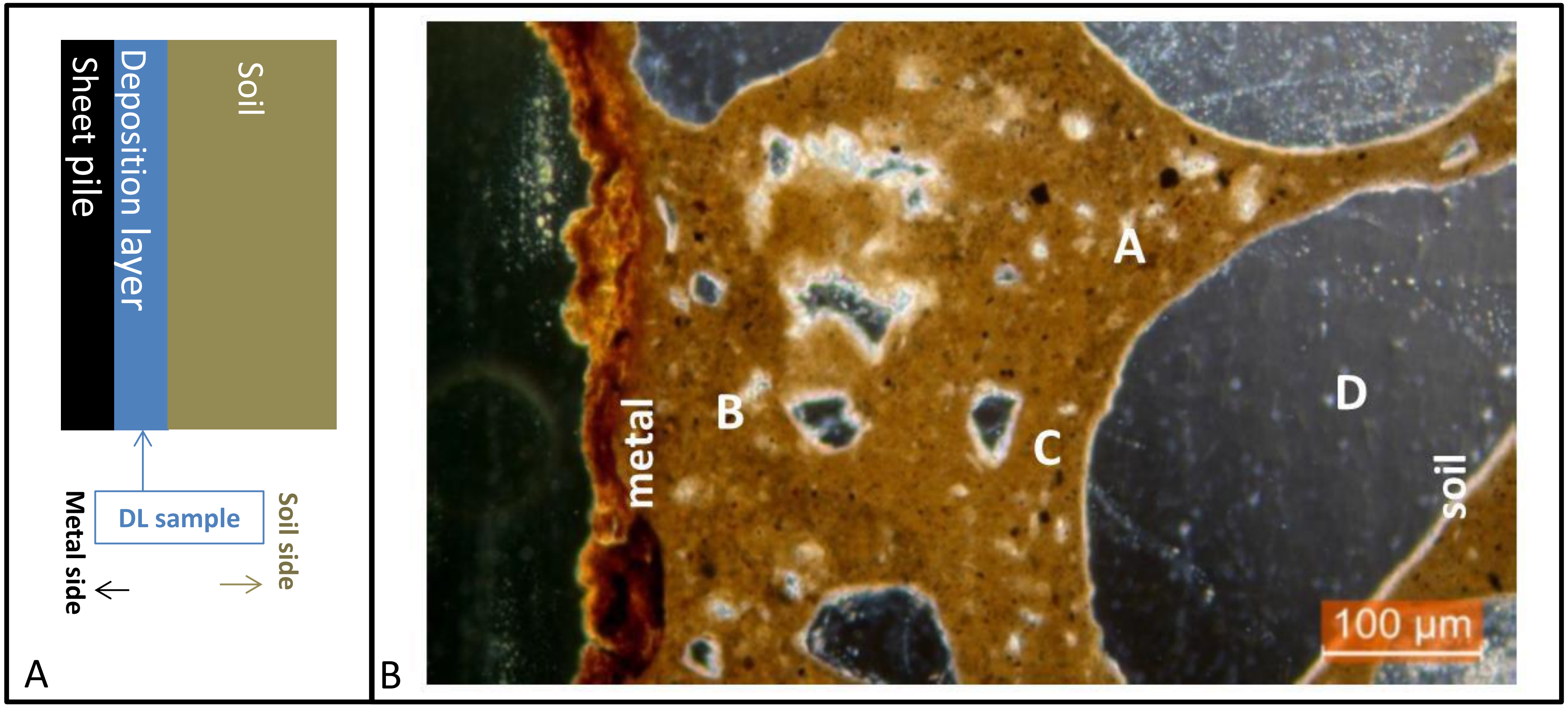

Figure S2. A: Schematic overview of the positioning of the DL sample. Only the deposition layer was looked at under the microscope. B: Phase contrast micrograph of a cross section of the deposition layer from the Westerkade site. A dense, iron rich, layer on the metal side and a more porous layer on the soil side are visible. Explanation of the letters within figureS2B: A: quarts mineral, B: dense layer, C: porous layer, D: sand grain

A. Archaeal classes

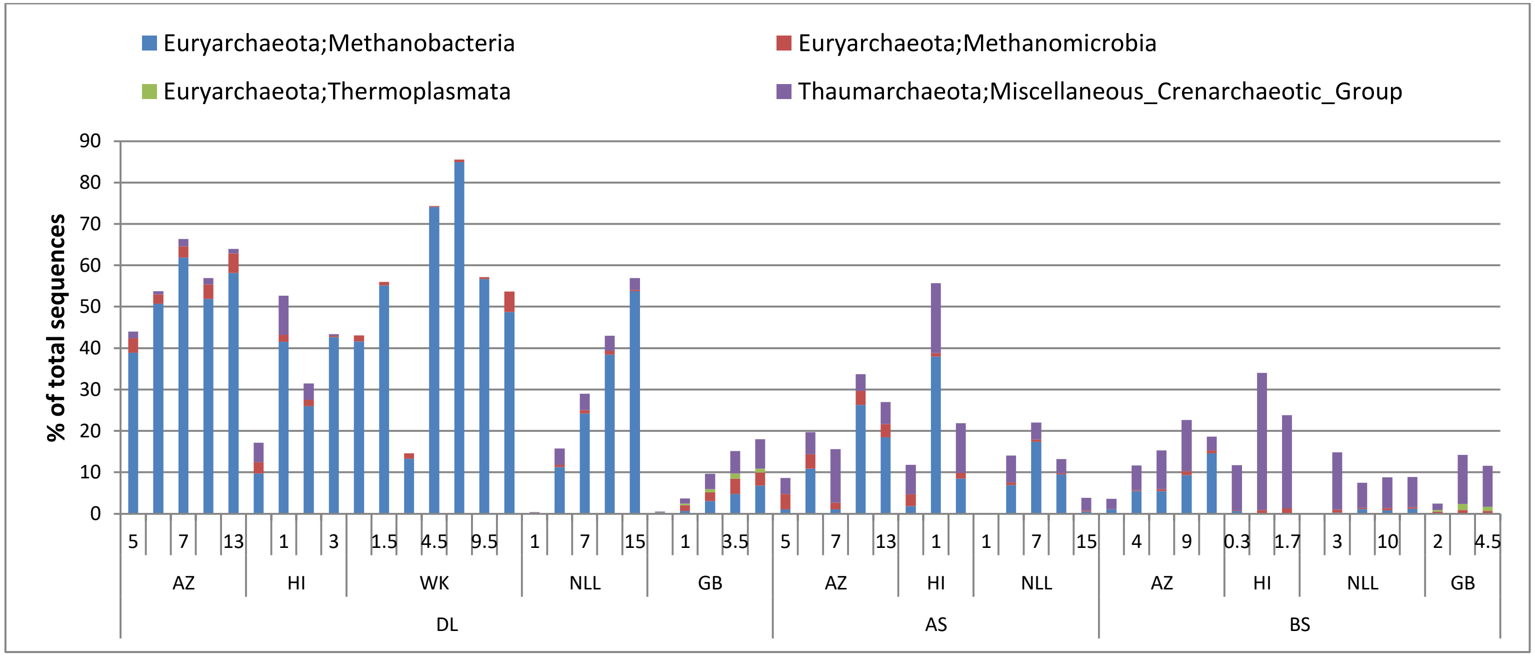

B. Proteobacterial classes

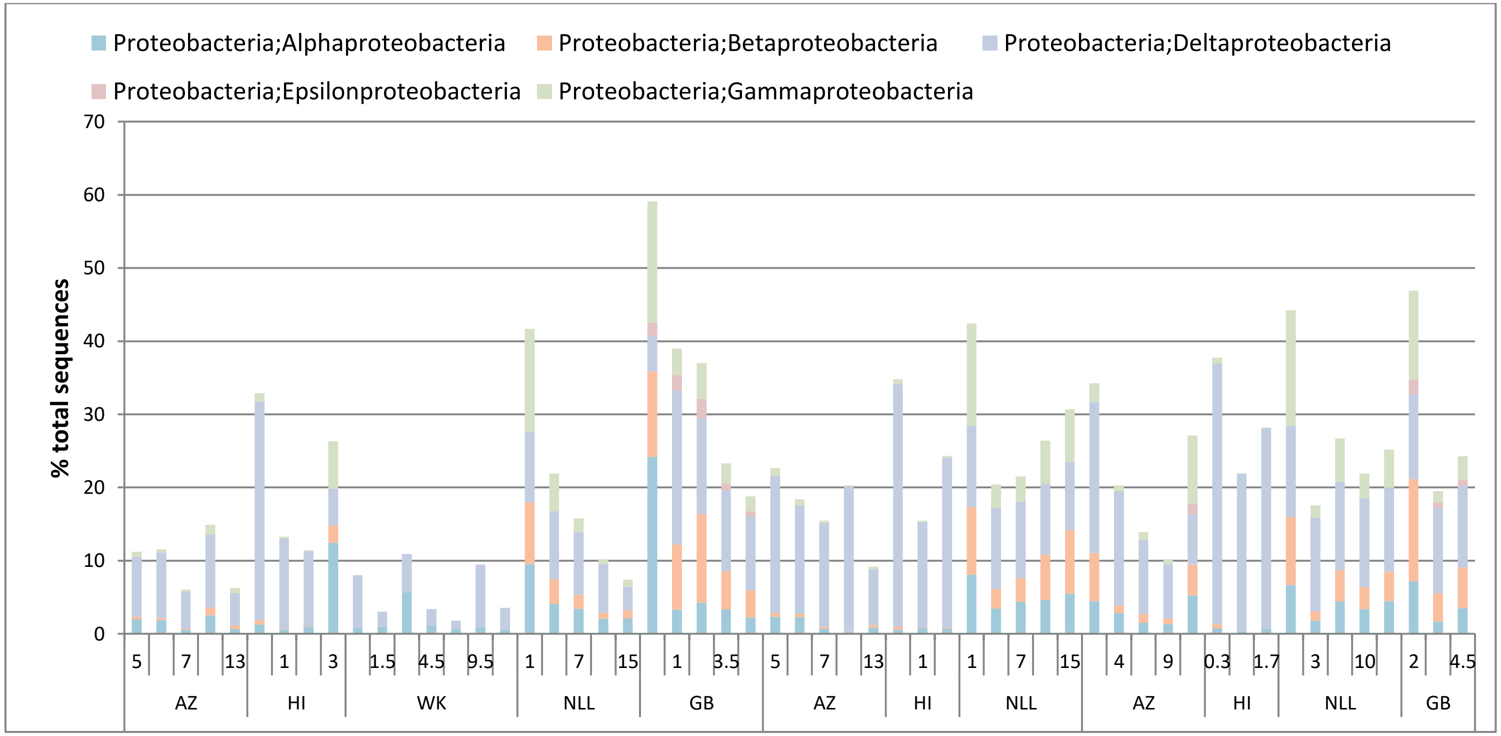

C. Nitrospira classes

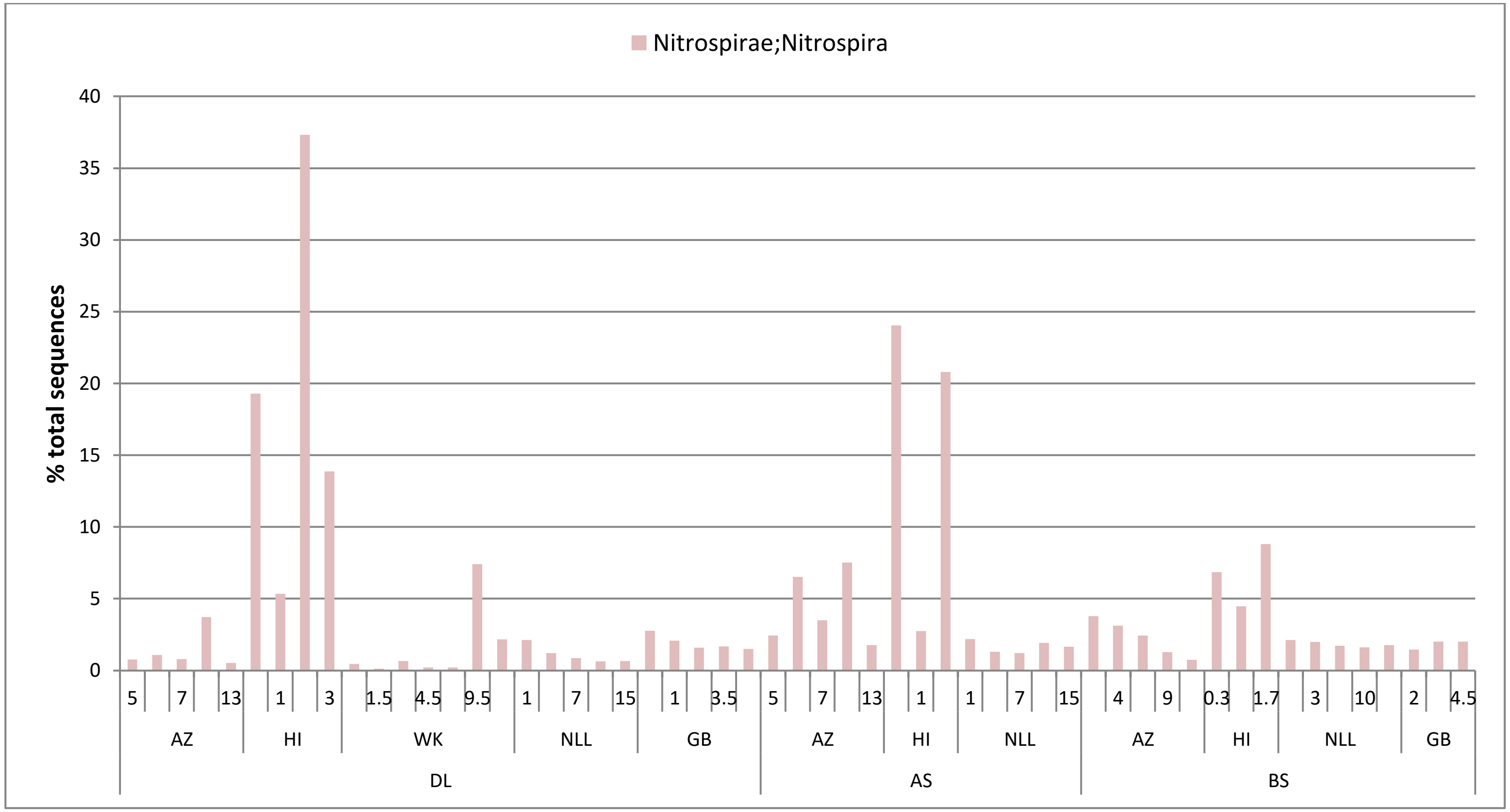

D. Acidobacterial and Actinobacterial classes

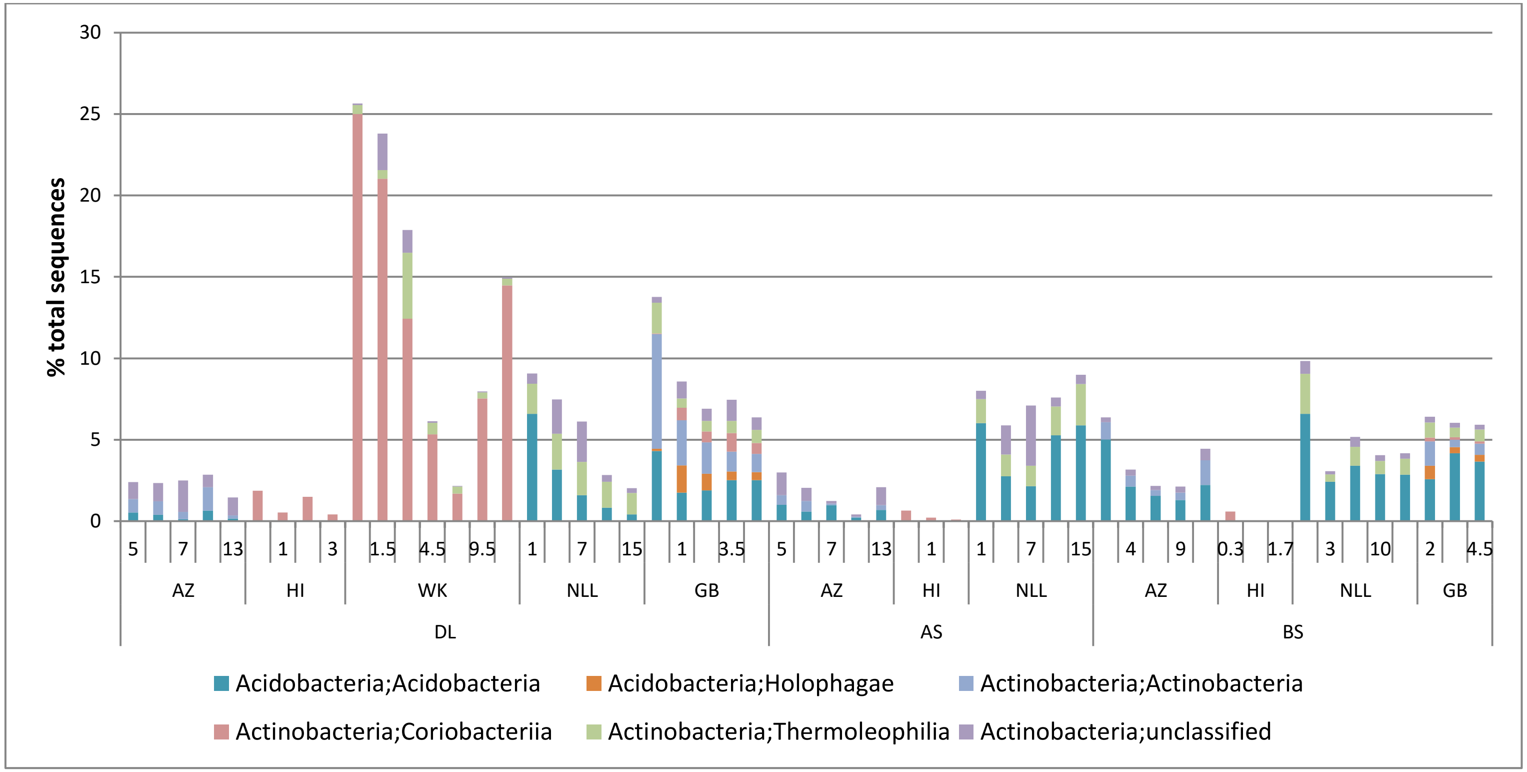

E. Chloroflexi classes

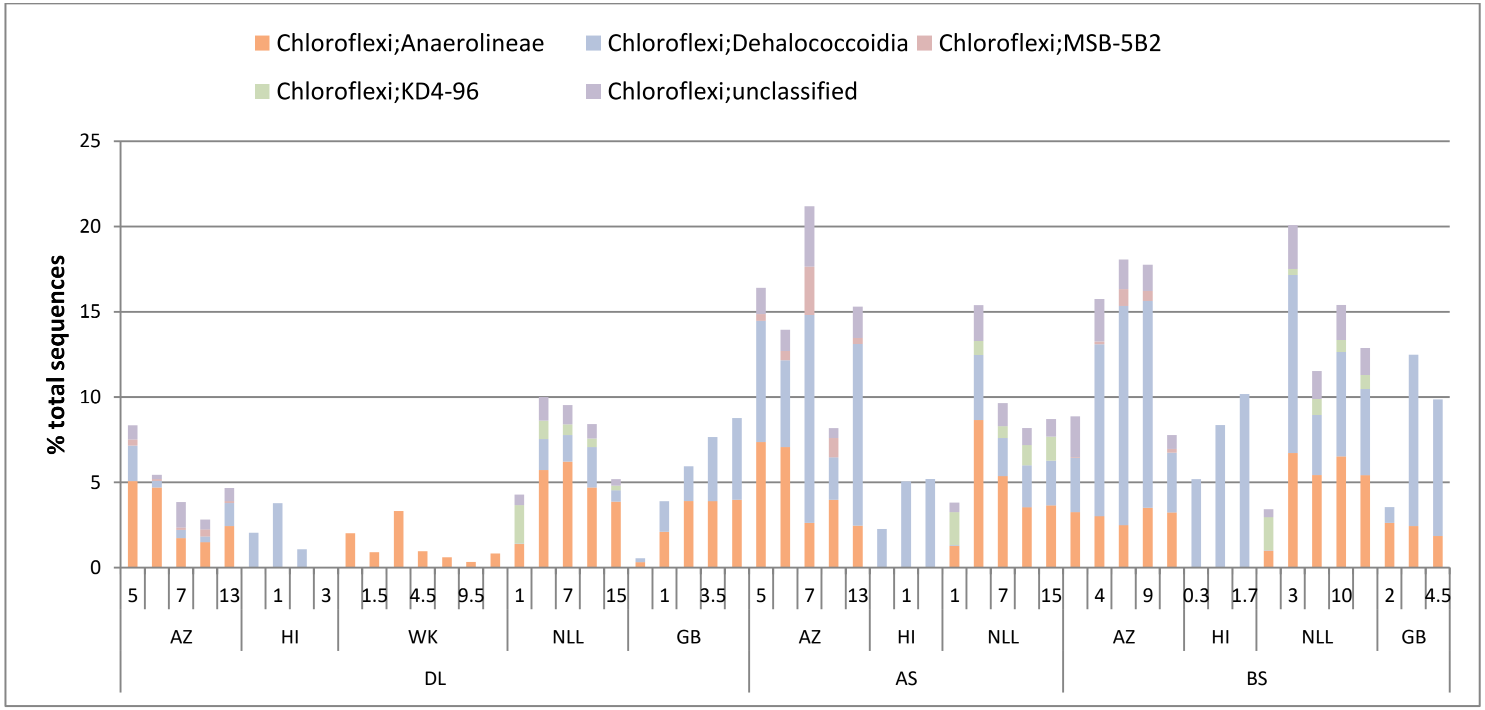

F. candidate division' classes

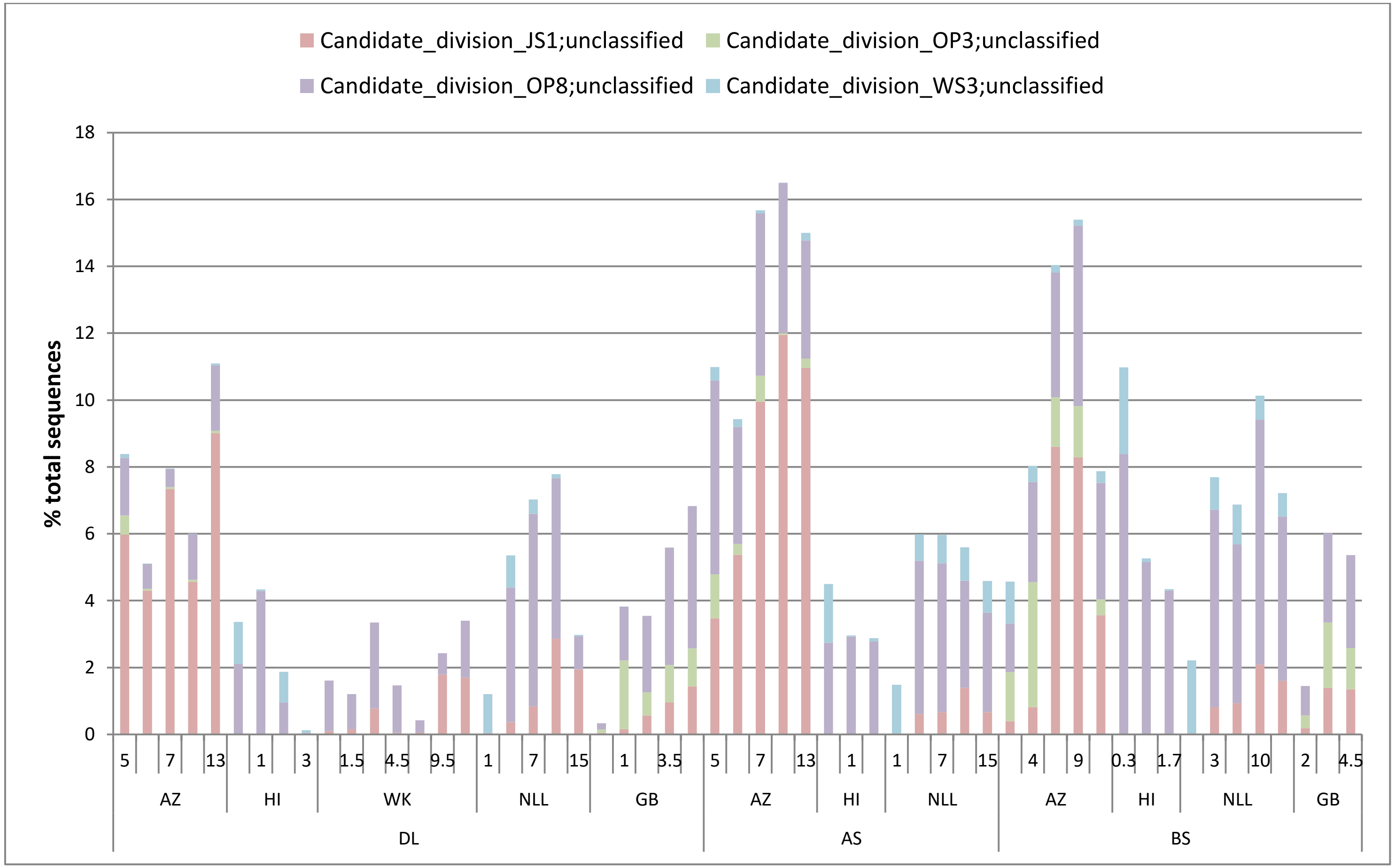

G. Bacteriodetes, Firmicutes, Planctomycetes and Gemmatimonadetes classes

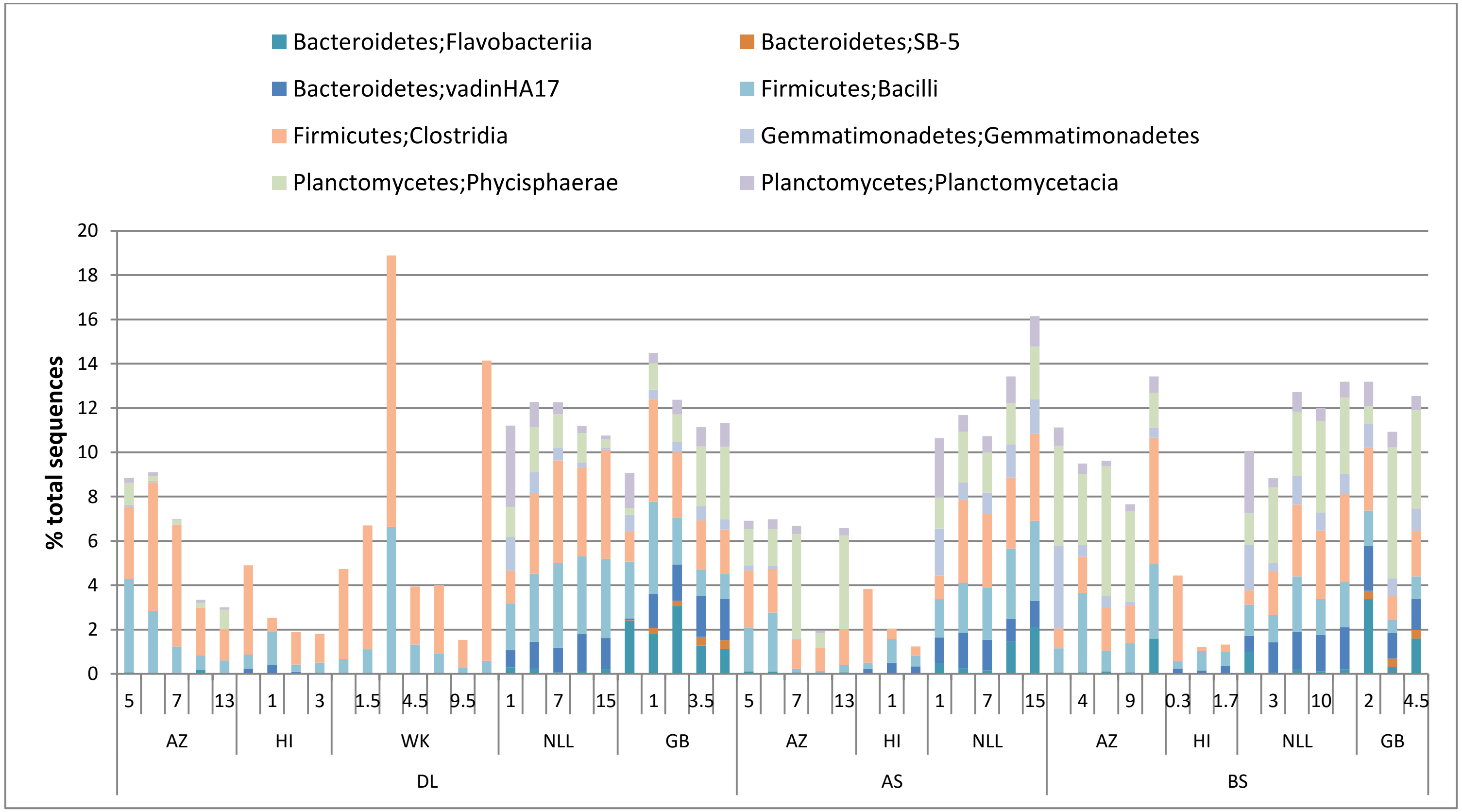

H. Spirochaetes and Verrucomicrobial classes and unclassified and unknown sequences

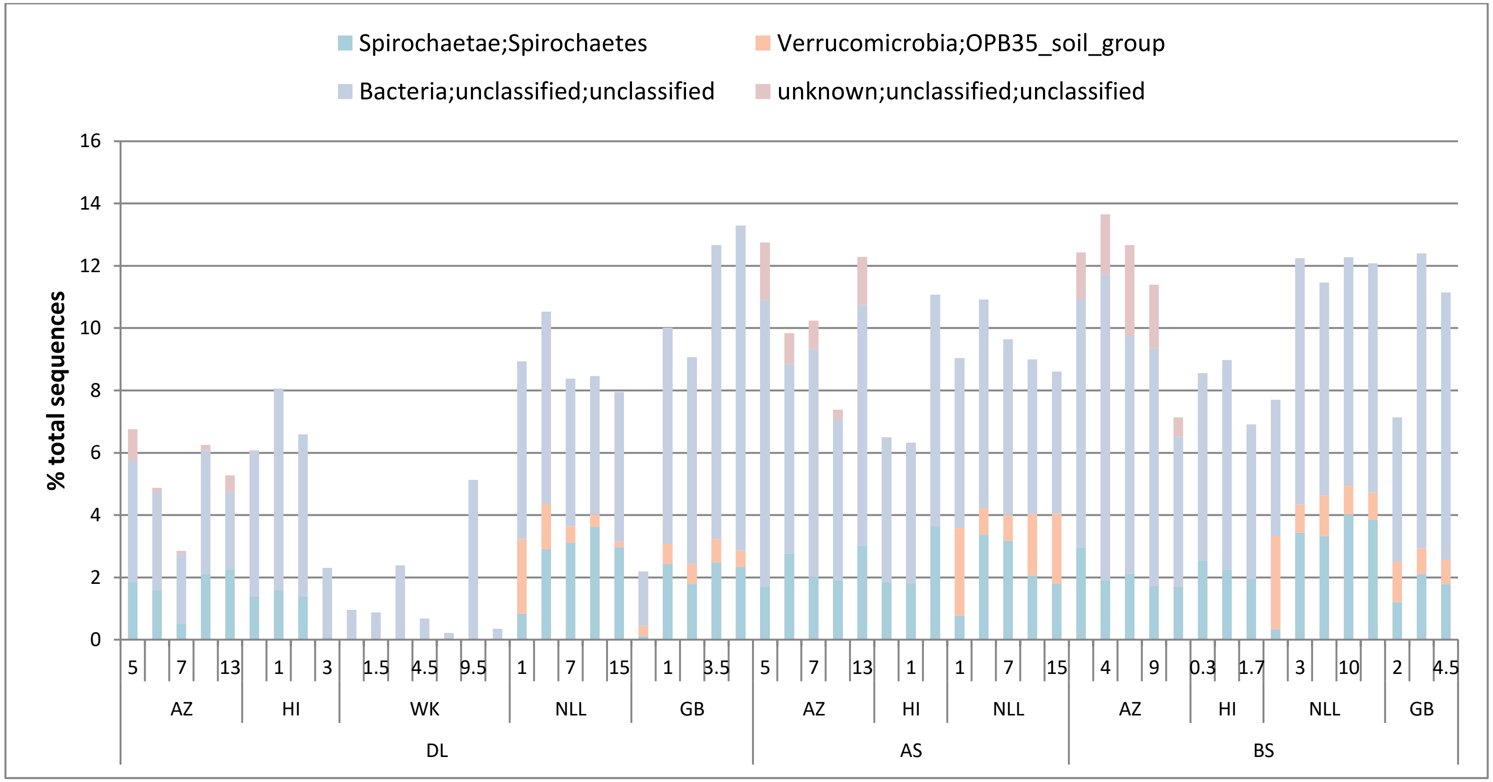

I. All Classes

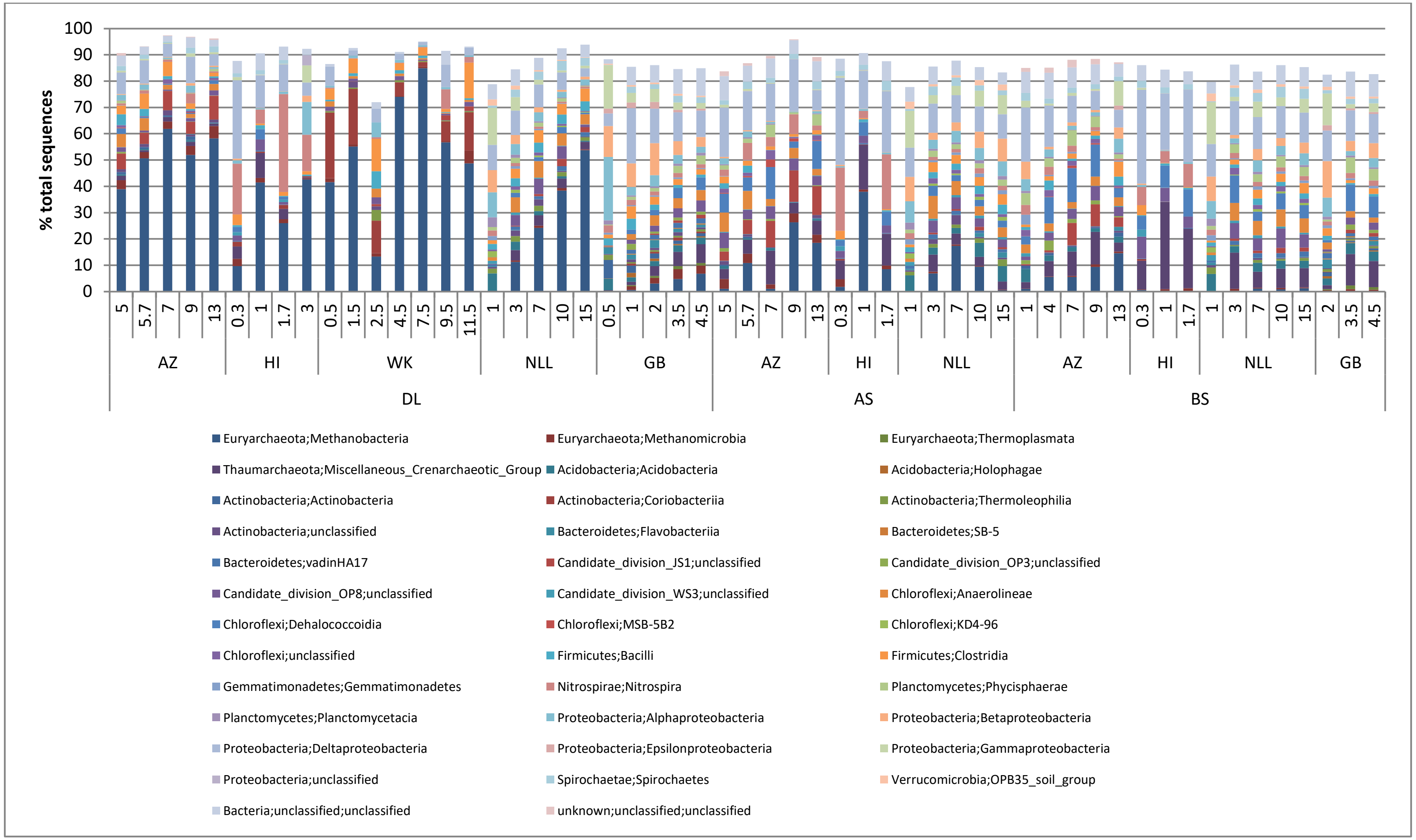

Supplementary Figure S3. Stacked diagrams of all the relative abundances of the different classes (averages, n=3). Only showing classes>2% in one sample or more.

A. Archaeal genera

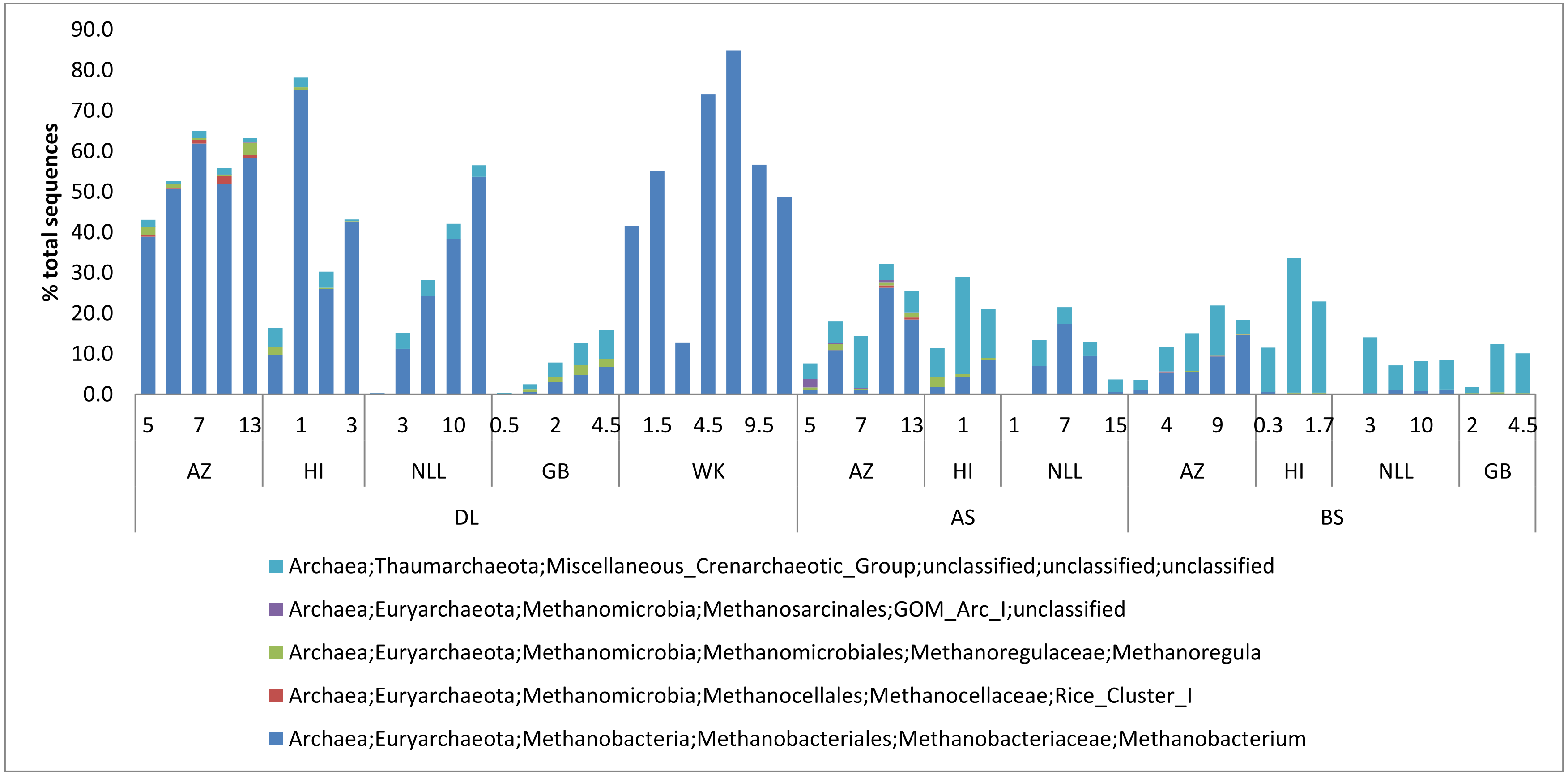

B. Alpha and Beta proteobacterial genera.

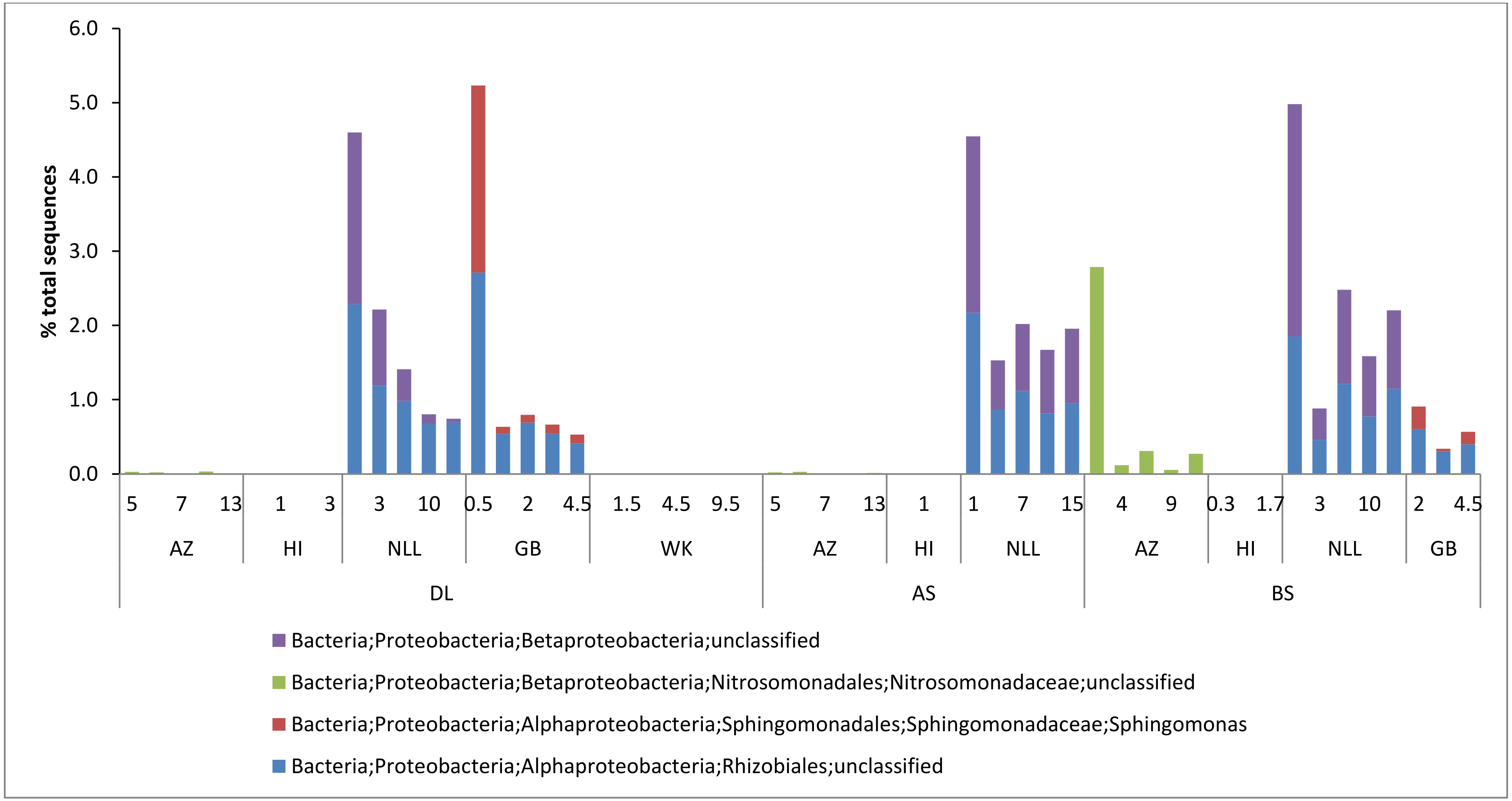

C. Deltaproteobacterial genera

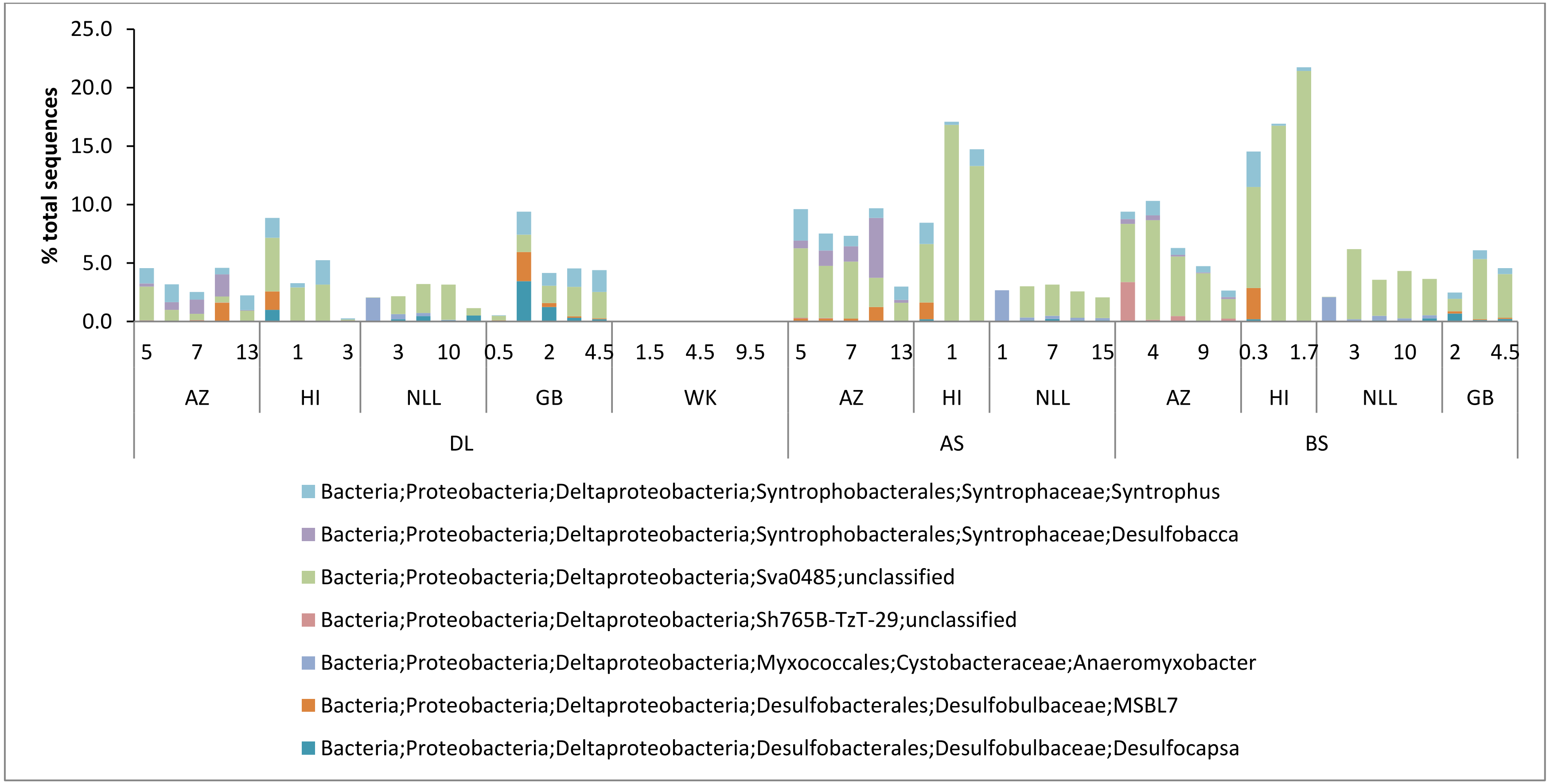

D. Gammaproteobacterial genera

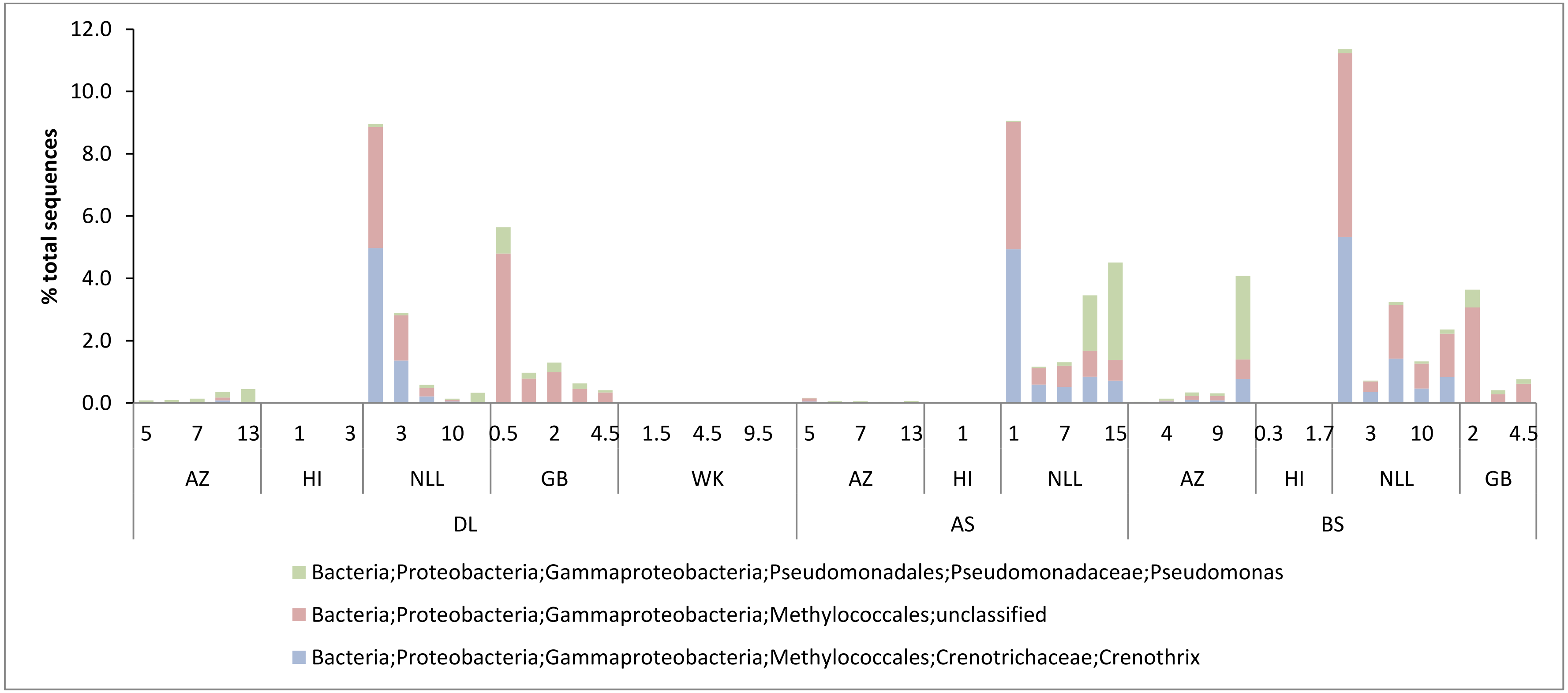

E. Nitrospira genera

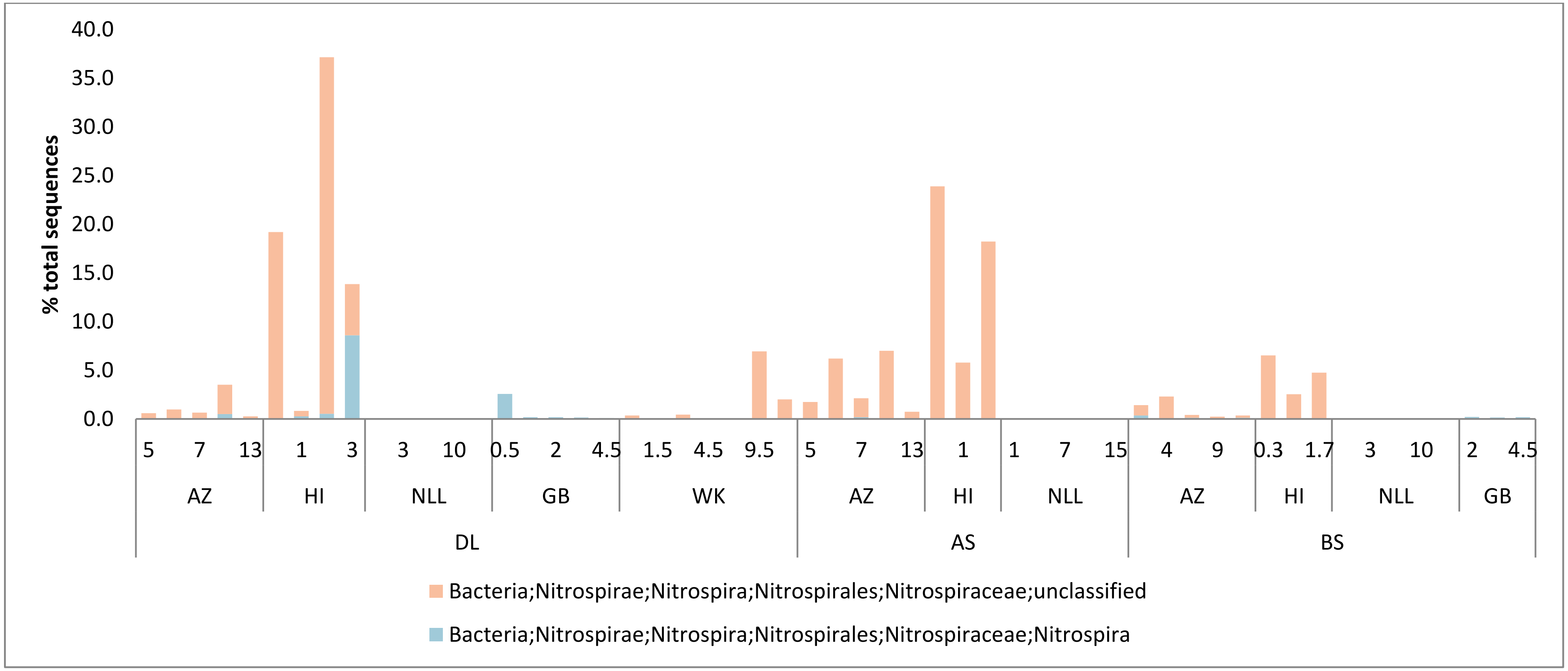

F. genera of Acino and Acidobacteria and Bacteriodetes and Candidate divisions.

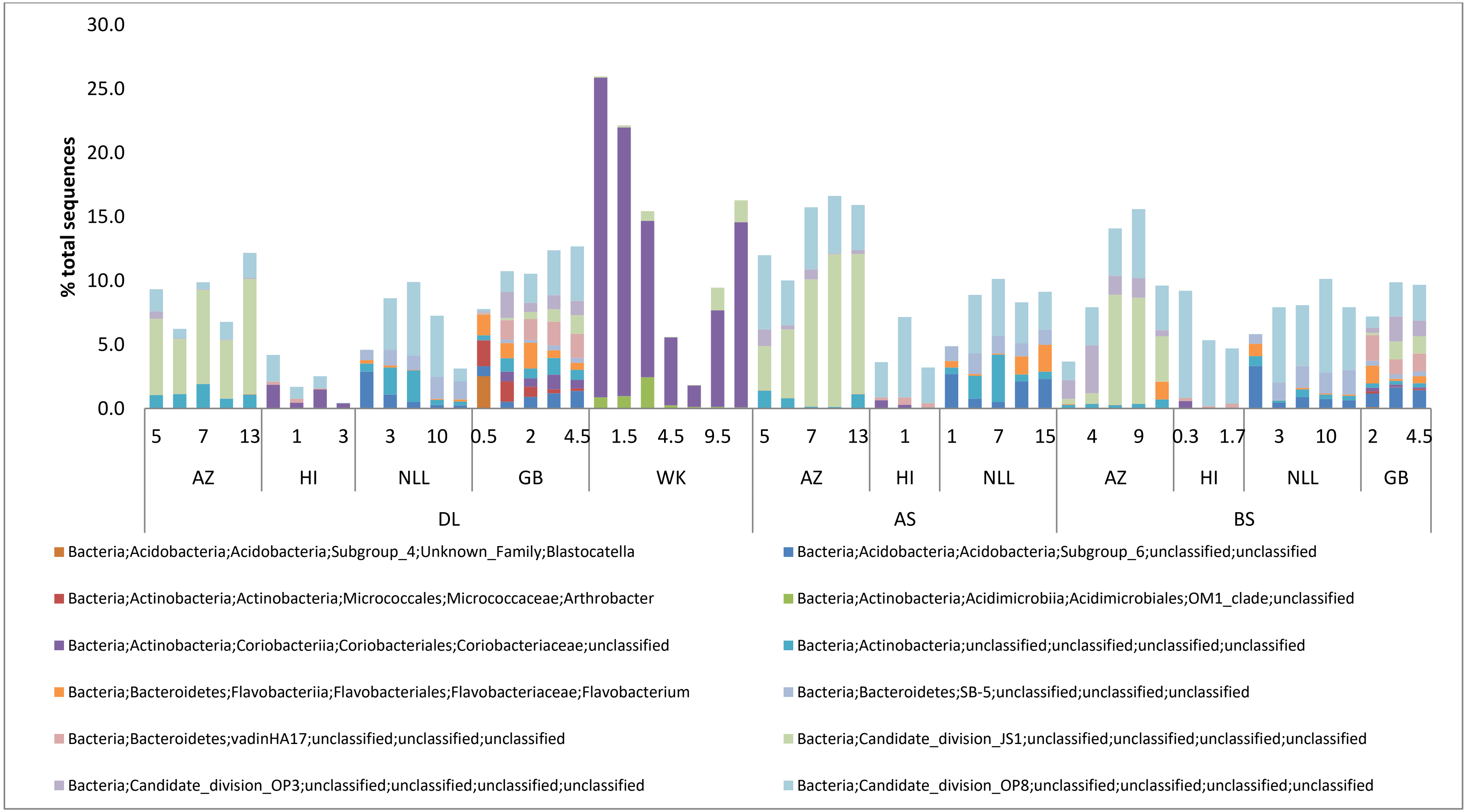

G. Classes of Chloroflexi, FIrmicutes, Gemmatimonadetes and Planctomycetes.

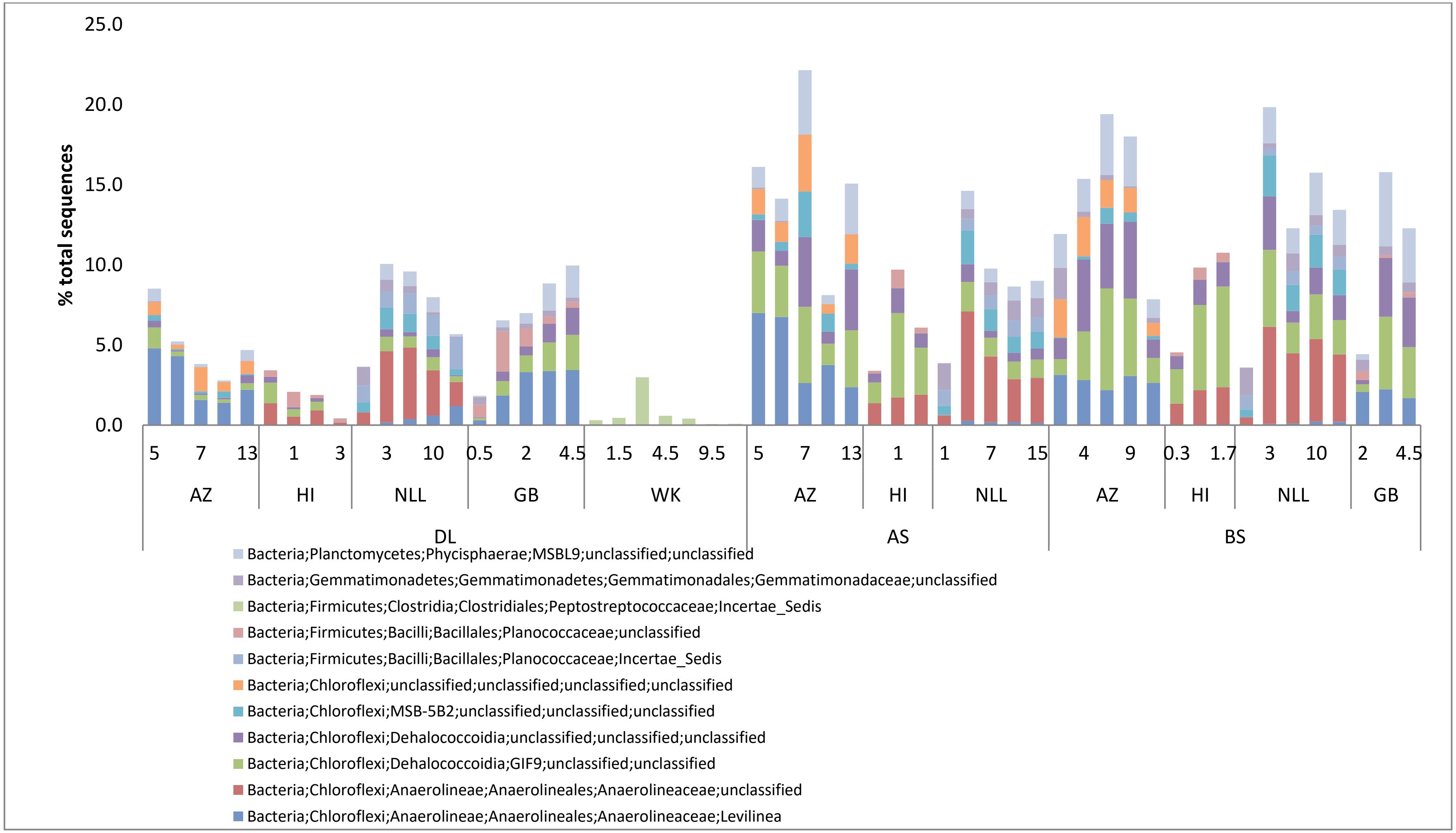

Supplementary Figure S4. Stacked diagrams of all the relative abundances of the different classes (averages, n=3). Only showing classes>2% in one sample or more.

Supplementary Table S4. Sample ID, treatment, total number of sequences and Good's coverage using DNA-based approach

Sample ID	Sample Type	Depth (m)	Total number of sequences	Good's coverage
**Amsterdamse Zeeburgertunnel**				
AZ12AA	BS	1	5642	0.99185
AZ12AB	BS	1	3262	0.98437
AZ12AC	BS	1	2441	0.97542
AZ12BA	BS	1	4064	0.98597
AZ12BB	BS	1	5703	0.99369
AZ12BC	BS	1	2168	0.97509
AZ12CA	BS	1	4226	0.99006
AZ12CB	BS	1	3461	0.98584
AZ12CC	BS	1	8808	0.99546
AZ1A2A	DP	5	2708	0.97747
AZ1A2B	DP	5	1852	0.96706
AZ1A2C	DP	5	3332	0.98109
AZ1BA	DP	5	5527	0.9886
AZ1BB	DP	5	5212	0.98599
AZ1BC	DP	5	2318	0.97325
AZ1C1A	AS	5	7844	0.99159
AZ1C1B	AS	5	3141	0.97867
AZ1C1C	AS	5	5473	0.98977
AZ1C2A	DP	5	886	0.9526
AZ1C2B	DP	5	1849	0.97891
AZ1C2C	DP	5	1304	0.96472
AZ22AA	BS	7	5626	0.99271
AZ22AB	BS	7	3968	0.98916
AZ22AC	BS	7	6461	0.99087
AZ22BA	BS	7	4560	0.98969
AZ22BB	BS	7	1502	0.9747
AZ22BC	BS	7	2340	0.9859
AZ22CA	BS	7	1455	0.97595
AZ22CB	BS	7	1781	0.98596
AZ22CC	BS	7	2349	0.98255
AZ2A1A	AS	5.7	1674	0.96535
AZ2A1B	AS	5.7	3038	0.97696
AZ2A1C	AS	5.7	3749	0.97893
AZ2A2A	DP	5.7	2102	0.97193
AZ2A2B	DP	5.7	1729	0.97629
AZ2A2C	DP	5.7	1580	0.97278

AZ2B1A	AS	5.7	2110	0.9654
AZ2B1B	AS	5.7	5926	0.98701
AZ2B1C	AS	5.7	4915	0.98454
AZ2B2A	DP	5.7	432	0.9213
AZ2B2B	DP	5.7	525	0.92571
AZ2B2C	DP	5.7	948	0.96097
AZ2C1A	AS	5.7	2613	0.97551
AZ2C1B	AS	5.7	4618	0.98506
AZ2C1C	AS	5.7	5276	0.98711
AZ2C2A	DP	5.7	2172	0.97836
AZ2C2B	DP	5.7	1692	0.97104
AZ2C2C	DP	5.7	908	0.95595
AZ3A1B	AS	7	3629	0.99421
AZ3A1C	AS	7	3336	0.99221
AZ3A2A	DP	7	1500	0.97933
AZ3A2B	DP	7	1111	0.9766
AZ3A2C	DP	7	744	0.97312
AZ3B1A	AS	7	2620	0.99084
AZ3B1B	AS	7	2105	0.98432
AZ3B1C	AS	7	2496	0.99159
AZ3B2A	DP	7	1466	0.97681
AZ3B2B	DP	7	1931	0.97566
AZ3B2C	DP	7	667	0.96702
AZ3C1A	AS	7	3329	0.99039
AZ3C1B	AS	7	1738	0.97296
AZ3C1C	AS	7	1933	0.9762
AZ3C2A	DP	7	1430	0.97273
AZ3C2B	DP	7	2419	0.98429
AZ42AA	BS	4	4831	0.98675
AZ42AB	BS	4	6292	0.9911
AZ42AC	BS	4	6376	0.992
AZ42BA	BS	4	4942	0.98988
AZ42BB	BS	4	8929	0.99474
AZ42BC	BS	4	6041	0.99073
AZ42CA	BS	4	4075	0.98479
AZ42CB	BS	4	3534	0.98246
AZ42CC	BS	4	1768	0.96041
AZ4A1A	AS	9	1938	0.98916
AZ4A1B	AS	9	2083	0.99232
AZ4A1C	AS	9	2412	0.98922
AZ4A2A	DP	9	1004	0.98606
AZ4A2B	DP	9	874	0.97941
AZ4A2C	DP	9	2124	0.98776
AZ4B1A	AS	9	1116	0.97491

AZ4B1B	AS	9	2642	0.98713
AZ4B1C	AS	9	3207	0.98909
AZ4B2A	DP	9	1464	0.97951
AZ4B2B	DP	9	804	0.96144
AZ4C1A	AS	9	1316	0.9734
AZ4C1B	AS	9	915	0.97596
AZ4C2A	DP	9	220	0.87273
AZ4C2B	DP	9	778	0.96401
AZ4C2C	DP	9	3073	0.98959
AZ4C3A	DP	9	1973	0.98834
AZ52AA	BS	9	584	0.94863
AZ52AB	BS	9	1274	0.98116
AZ52AC	BS	9	1546	0.98124
AZ52BA	BS	9	2122	0.97455
AZ52BB	BS	9	3161	0.98671
AZ52BC	BS	9	5400	0.99259
AZ52CA	BS	9	5797	0.98999
AZ52CB	BS	9	6168	0.99092
AZ52CC	BS	9	2428	0.97446
AZ5A1A	AS	13	3855	0.98859
AZ5A1B	AS	13	4078	0.98847
AZ5A2A	DP	13	1795	0.98552
AZ5A2A5	DP	13	1235	0.97895
AZ5A2B	DP	13	1578	0.98859
AZ5A2B5	DP	13	1138	0.98067
AZ5A2C	DP	13	1469	0.98026
AZ5A2C5	DP	13	961	0.97294
AZ5B1A	AS	13	3279	0.98719
AZ5B1B	AS	13	2190	0.97717
AZ5B1C	AS	13	1028	0.96595
AZ5B2B	DP	13	1991	0.97338
AZ5B2C	DP	13	1074	0.94879
AZ5C1A	AS	13	867	0.96194
AZ5C1B	AS	13	3567	0.98879
AZ5C1C	AS	13	2436	0.97989
AZ5C2A	DP	13	1116	0.96953
AZ5C2B	DP	13	1388	0.97695
AZ5C2C	DP	13	2459	0.97275
AZ63AA	BS	13	5223	0.98775
AZ63AB	BS	13	4955	0.99233
AZ63AC	BS	13	5187	0.99132
AZ63BA	BS	13	1484	0.95148
AZ63BB	BS	13	2202	0.97275
AZ63BC	BS	13	6970	0.98938

AZ63CA	BS	13	1500	0.96067
AZ63CB	BS	13	1228	0.94055
AZ63CC	BS	13	3199	0.98281
**Giessenburg**				
GB1LA	DP	2	5337	0.982
GB1LB	DP	2	4805	0.982
GB1LC	DP	2	6432	0.986
GB1PL2LB	DP	2	5984	0.979
GB1PL2LC	DP	2	5459	0.979
GB1PL2MB	DP	2	3018	0.967
GB1PL2MC	DP	2	8242	0.99
GB1PL2RB	DP	2	5243	0.98
GB1PL2RC	DP	2	9045	0.99
GB1RA	DP	2	4487	0.971
GB1RB	DP	2	6556	0.982
GB1RC	DP	2	5618	0.981
GB2LA	DP	3.5	3809	0.968
GB2LB	DP	3.5	10484	0.99
GB2LC	DP	3.5	4695	0.974
GB2MA	DP	3.5	10868	0.991
GB2MB	DP	3.5	4432	0.98
GB2MC	DP	3.5	7195	0.986
GB2PL2LA	DP	3.5	12588	0.993
GB2PL2LB	DP	3.5	7003	0.986
GB2PL2LC	DP	3.5	8105	0.988
GB2PL2MA	DP	3.5	3226	0.97
GB2PL2MB	DP	3.5	1240	0.945
GB2PL2MC	DP	3.5	5332	0.98
GB2PL2RA	DP	3.5	3139	0.973
GB2PL2RB	DP	3.5	4465	0.984
GB2PL2RC	DP	3.5	2430	0.968
GB2RA	DP	3.5	8930	0.989
GB2RB	DP	3.5	5415	0.977
GB2RC	DP	3.5	4173	0.974
GB3LA	DP	4.5	2947	0.958
GB3LB	DP	4.5	4261	0.973
GB3LC	DP	4.5	3092	0.966
GB3MA1	DP	4.5	3509	0.982
GB3MA2	DP	4.5	5974	0.987
GB3MB	DP	4.5	3421	0.972
GB3PL2LA	DP	4.5	2820	0.963
GB3PL2LB	DP	4.5	8455	0.989
GB3PL2LC	DP	4.5	8551	0.986
GB3PL2MB	DP	4.5	3652	0.979

GB3PL2MC	DP	4.5	3638	0.978
GB3PL2RA	DP	4.5	2112	0.959
GB3PL2RB	DP	4.5	6159	0.986
GB3PL2RC	DP	4.5	5194	0.98
GBBLA	DP	1	3510	0.981
GBBLB	DP	1	5455	0.987
GBBLC	DP	1	6575	0.988
GBBMA	DP	1	6259	0.985
GBBMB	DP	1	6634	0.986
GBBMC	DP	1	8101	0.989
GBBRA	DP	1	9961	0.991
GBBRB	DP	1	7361	0.984
GBBRC	DP	1	8908	0.991
GBBS1A	BS	2	11147	0.993
GBBS1B	BS	2	5380	0.978
GBBS1C	BS	2	10274	0.992
GBBS2A	BS	3.5	4863	0.986
GBBS2B	BS	3.5	3014	0.979
GBBS2C	BS	3.5	2639	0.976
GBBS3A	BS	4.5	4427	0.977
GBBS3B	BS	4.5	4826	0.973
GBBS3C	BS	4.5	6814	0.985
GBSH1A	BS	2	4354	0.975
GBSH1B	BS	2	11029	0.992
GBSH1C	BS	2	10842	0.993
GBSH2A	BS	3.5	12058	0.992
GBSH2B	BS	3.5	9234	0.988
GBSH2C	BS	3.5	9559	0.989
GBSH3A	BS	4.5	10130	0.991
GBSH3B	BS	4.5	11372	0.991
GBSH3C	BS	4.5	9152	0.99
**Hollandse Ijsselkade**				
HI_A_1BA	DP	0.3	7637	0.995
HI_A_1BB	DP	0.3	1277	0.973
HI_A_1BC	DP	0.3	1559	0.979
HI_A_1TA	AS	0.3	9619	0.996
HI_A_1TB	AS	0.3	10175	0.996
HI_A_1TC	AS	0.3	7085	0.994
HI_A_2BA	DP	1	10954	0.996
HI_A_2BB	DP	1	11561	0.996
HI_A_2BC	DP	1	10571	0.996
HI_A_2TA	AS	1	8941	0.996
HI_A_2TB	AS	1	9704	0.997
HI_A_2TC	AS	1	9334	0.996

HI_A_3BA	DP	1.7	10751	0.996
HI_A_3BB	DP	1.7	17327	0.998
HI_A_3BC	DP	1.7	18104	0.998
HI_A_3TA	AS	1.7	5255	0.995
HI_A_3TB	AS	1.7	12072	0.997
HI_A_3TC	AS	1.7	12121	0.998
HI_A_4A	DP	3	13215	0.997
HI_A_4B	DP	3	19284	0.997
HI_A_4C	DP	3	3056	0.986
HI_BULK_1A	BS	0.3	7704	0.994
HI_BULK_1B	BS	0.3	11176	0.997
HI_BULK_1C	BS	0.3	10803	0.996
HI_BULK_2A	BS	1	8982	0.996
HI_BULK_2C	BS	1	18037	0.998
HI_BULK_3A	BS	1.7	5091	0.995
HI_B_1BA	DP	0.3	3682	0.991
HI_B_1BB	DP	0.3	11384	0.996
HI_B_1BC	DP	0.3	10178	0.996
HI_B_1TA	AS	0.3	13532	0.997
HI_B_1TB	AS	0.3	4266	0.991
HI_B_1TC	AS	0.3	7490	0.995
HI_B_2BA	DP	1	5841	0.994
HI_B_2BB	DP	1	54380	0.999
HI_B_2BC	DP	1	64422	1
HI_B_2TA	AS	1	4627	0.993
HI_B_2TB	AS	1	7546	0.994
HI_B_2TC	AS	1	6023	0.993
HI_B_3BA	DP	1.7	11994	0.997
HI_B_3BB	DP	1.7	87	0.92
HI_B_3BC	DP	1.7	9546	0.995
HI_B_3TA	AS	1.7	6094	0.994
HI_B_3TB	AS	1.7	11667	0.996
HI_B_3TC	AS	1.7	10665	0.995
HI_B_4A	DP	3	6509	0.994
HI_B_4B	DP	3	7489	0.996
HI_B_4C	DP	3	6677	0.996
**Nieuwlekkerland**				
NLL1AS1	DP	15	153	0.784
NLL1AS2	DP	15	123	0.724
NLL1AS3	DP	15	296	0.818
NLL1BS1	DP	15	180	0.739
NLL1BS2	DP	15	308	0.792
NLL1BS3	DP	15	375	0.795
NLL1BULKS1	BS	15	6695	0.987

NLL1BULKS2	BS	15	4895	0.981
NLL1BULKS3	BS	15	2402	0.963
NLL1CS1	AS	15	213	0.84
NLL1CS2	AS	15	521	0.856
NLL1CS3	AS	15	218	0.817
NLL1PL2S1	DP	15	245	0.767
NLL1PL2S2	DP	15	159	0.774
NLL1PL2S3	DP	15	128	0.773
NLL22AS1	DP	10	166	0.729
NLL22AS2	DP	10	443	0.86
NLL22AS3	DP	10	174	0.747
NLL22BS1	DP	10	1617	0.949
NLL22BS2	DP	10	3225	0.976
NLL22BS3	DP	10	3397	0.973
NLL22CS1	DP	10	900	0.892
NLL22CS2	DP	10	860	0.897
NLL22CS3	DP	10	745	0.89
NLL2A2S1	AS	10	3051	0.984
NLL2A2S2	AS	10	5748	0.993
NLL2A2S3	AS	10	5918	0.993
NLL2B2S1	AS	10	6987	0.985
NLL2B2S2	AS	10	9782	0.991
NLL2B2S3	AS	10	14470	0.995
NLL2BULKS1	BS	10	4449	0.98
NLL2BULKS2	BS	10	6759	0.986
NLL2BULKS3	BS	10	3753	0.975
NLL2C2S1	AS	10	5550	0.985
NLL2C2S2	AS	10	5578	0.98
NLL2C2S3	AS	10	6172	0.981
NLL2PL2S1	DP	10	618	0.859
NLL2PL2S2	DP	10	1857	0.941
NLL2PL2S3	DP	10	1735	0.954
NLL2SS1	AS	10	4322	0.974
NLL2SS2	AS	10	993	0.932
NLL2SS3	AS	10	410	0.888
NLL3AS1	DP	7	2624	0.959
NLL3AS2	DP	7	3052	0.97
NLL3AS3	DP	7	2219	0.958
NLL3BS1	DP	7	1947	0.957
NLL3BS2	DP	7	2340	0.962
NLL3BS3	DP	7	1843	0.946
NLL3BULKS1	BS	7	4713	0.983
NLL3BULKS2	BS	7	6334	0.985
NLL3BULKS3	BS	7	4427	0.981

NLL3CS1	DP	7	2050	0.957
NLL3CS2	DP	7	3178	0.972
NLL3CS3	DP	7	2954	0.964
NLL3GS1	AS	7	3722	0.974
NLL3GS2	AS	7	3513	0.972
NLL3GS3	AS	7	3318	0.969
NLL3PL2S1	DP	7	552	0.833
NLL3PL2S2	DP	7	755	0.883
NLL3PL2S3	DP	7	994	0.904
NLL4AS1	DP	3	2756	0.963
NLL4AS2	DP	3	3198	0.971
NLL4AS3	DP	3	3585	0.979
NLL4BS1	DP	3	3116	0.97
NLL4BS2	DP	3	2818	0.967
NLL4BS3	DP	3	2194	0.954
NLL4BULKS1	BS	3	2699	0.972
NLL4BULKS2	BS	3	4229	0.982
NLL4BULKS3	BS	3	4671	0.977
NLL4CS16	DP	3	3740	0.976
NLL4CS17	DP	3	3149	0.971
NLL4CS26	DP	3	6351	0.986
NLL4CS27	DP	3	5280	0.984
NLL4CS36	DP	3	1974	0.948
NLL4CS37	DP	3	1682	0.944
NLL4GS1	AS	3	3345	0.97
NLL4GS2	AS	3	5114	0.983
NLL4GS3	AS	3	9767	0.992
NLL4PL2S2	DP	3	3379	0.97
NLL4PL2S3	DP	3	5006	0.979
NLL5AS1	DP	1	5118	0.987
NLL5AS2	DP	1	6445	0.991
NLL5AS3	DP	1	6764	0.991
NLL5BS1	DP	1	6600	0.989
NLL5BS2	DP	1	3706	0.976
NLL5BS3	DP	1	3782	0.982
NLL5BULKS1	BS	1	5555	0.987
NLL5BULKS2	BS	1	4813	0.985
NLL5BULKS3	BS	1	4415	0.984
NLL5CS1	DP	1	1785	0.959
NLL5CS2	DP	1	4595	0.987
NLL5CS3	DP	1	5147	0.988
NLL5GS1	AS	1	2809	0.974
NLL5GS2	AS	1	4902	0.986
NLL5GS3	AS	1	4955	0.984

NLLA1S1	AS	15	5697	0.991
NLLA1S2	AS	15	4967	0.989
NLLA1S3	AS	15	8456	0.995
NLLB1S1	AS	15	8769	0.99
NLLB1S2	AS	15	2634	0.954
NLLB1S3	AS	15	3569	0.966
NLLC1S1	DP	15	7228	0.985
NLLC1S2	DP	15	7742	0.989
NLLC1S3	DP	15	6202	0.985
**Westerkade**				
WK_1_AA	DP	11.5	3503	0.982
WK_1_AB	DP	11.5	8150	0.988
WK_1_AC	DP	11.5	13833	0.994
WK_1_BA	DP	11.5	17706	0.997
WK_1_BB	DP	11.5	17299	0.997
WK_1_BC	DP	11.5	16043	0.996
WK_1_CA	DP	11.5	8586	0.993
WK_2_AA	DP	9.5	9310	0.994
WK_2_AB	DP	9.5	6259	0.988
WK_2_AC	DP	9.5	4548	0.989
WK_2_BA	DP	9.5	9380	0.993
WK_2_BC	DP	9.5	6444	0.99
WK_3_AB	DP	7.5	8091	0.995
WK_3_AC	DP	7.5	13258	0.996
WK_3_BA	DP	7.5	8059	0.991
WK_3_BB	DP	7.5	15546	0.995
WK_3_BC	DP	7.5	7375	0.99
WK_3_CA	DP	7.5	8899	0.992
WK_3_CB	DP	7.5	11025	0.993
WK_3_CC	DP	7.5	20569	0.996
WK_4_AA	DP	4.5	12910	0.994
WK_4_AB	DP	4.5	4977	0.983
WK_4_AC	DP	4.5	16380	0.995
WK_4_BA	DP	4.5	7574	0.99
WK_4_BB	DP	4.5	3229	0.979
WK_4_BC	DP	4.5	17724	0.996
WK_4_CA	DP	4.5	8130	0.99
WK_4_CB	DP	4.5	2575	0.976
WK_4_CC	DP	4.5	13211	0.995
WK_5_AA	DP	2.5	7554	0.988
WK_5_BA	DP	2.5	15694	0.997
WK_5_BB	DP	2.5	18468	0.997
WK_5_BC	DP	2.5	7289	0.991
WK_5_CA	DP	2.5	12940	0.995

WK_5_CB	DP	2.5	10026	0.993
WK_5_CC	DP	2.5	11154	0.995
WK_6_AA	DP	1.5	20412	0.997
WK_6_AB	DP	1.5	26213	0.997
WK_6_AC	DP	1.5	23287	0.997
WK_6_BA	DP	1.5	15867	0.995
WK_6_BB	DP	1.5	14564	0.996
WK_6_BC	DP	1.5	13250	0.995
WK_6_CA	DP	1.5	16277	0.994
WK_7_AA	DP	0.5	16601	0.997
WK_7_AB	DP	0.5	21165	0.997
WK_7_AC	DP	0.5	16105	0.995
WK_7_BA	DP	0.5	13559	0.995
WK_7_BB	DP	0.5	18853	0.996
WK_7_BC	DP	0.5	16833	0.995
